# Cladistic assessment of subtribal affinities within the tribe Moriomorphini with description of *Rossjoycea glacialis*, gen. n. and sp. n. from the South Island, and revision of *Meonochilus* Liebherr and Marris from the North Island, New Zealand (Coleoptera, Carabidae)

**DOI:** 10.3897/zookeys.147.1898

**Published:** 2011-11-16

**Authors:** James K. Liebherr

**Affiliations:** 1 Department of Entomology, John H. and Anna B. Comstock Hall, Cornell University, Ithaca, NY 14853-2601, USA

**Keywords:** Allopatric speciation, biogeography, classification, genitalic evolution, Psydrinae, Psydrini

## Abstract

Phylogenetic relationships within the tribe Moriomorphini Sloane, 1890 are analyzed cladistically based on 75 morphological characters and 21 ingroup terminal taxa rooted at a *Trechus obtusus* Erichson outgroup. Based on the resultant cladistic relationships, two subtribes–Moriomorphina and Amblytelina Blackburn, 1892–are recognized, with the following new synonymies proposed: Meonides Sloane, 1898 = Amblytelina (NEW SYNONYMY); Tropopterides Sloane, 1898 = Amblytelina (NEW SYNONYMY); Mecyclothoracitae Jeannel, 1940 = Amblytelina (NEW SYNONYMY). Monophyly of Moriomorphina is based on presence of elongate, parallel-sided and glabrous to nearly glabrous male parameres, whereas Amblytelina are defined most broadly by possession of conchoid parameres with narrowed, setose apices, subtending a clade defined by a more derived parameral configuration whereby elongate styloid parameres terminate in a whip-like apical extension. Representatives of all New Zealand moriomorphine genera are included in the analysis, with cladistic results necessitating description of *Rossjoycea glacialis*, **gen. n.** and **sp. n.**, known from a single locality near the Franz Josef Glacier, Westland, South Island, New Zealand. Monophyly of *Meonochilus* Liebherr and Marris, 2009 is demonstrated, and its six species are taxonomically revised: *Meonochilus amplipennis* (Broun), *Meonochilus eplicatus* (Broun), *Meonochilus placens* (Broun), *Meonochilus bellorum*, **sp. n.**, *Meonochilus rectus*, **sp. n.**, and *Meonochilus spiculatus*, **sp. n.** Geographic restriction of *Meonochilus* to the North Island of New Zealand, coupled with its sister-group status to an Australian-based *Amblytelus* Erichson-*Mecyclothorax* Sharp clade reinforce the interpretation that *Meonochilus* was isolated in New Zealand by vicariance along the Norfolk Ridge, subsequent to New Zealand’s initial Cretaceous isolation from Tasmania and southeastern Australia via opening of the Tasman Sea.

## Introduction

The Austral disjunct tribe Moriomorphini has only recently been recognized as a natural group (Baehr 1998; Maddison et al. 1999). Mounting evidence for the close relationships of the five strictly Austral tribes represented in Moore’s (1963) worldwide treatment of his subfamily Psydrinae begs the question of phylogenetic relationships among the constituent Austral taxa. This paper addresses that question, and establishes the first morphologically-based cladistic hypothesis for species-level terminals representing genera considered to belong to the Austral “Psydrini” (Maddison et al. 1999). The cladistic relationships derived from this analysis provide the basis for grouping genera previously arrayed among five depauperate family-level taxa into two subtribes broadly defined by derivations of the male genitalia. The analysis also provides a sound basis for lower-level taxonomy. One terminal in the analysis, represented by a unique female specimen collected by Rowan Emberson on a scree field adjacent to an upper arm of the Franz Josef Glacier, Westland, New Zealand, is shown to exhibit a cladistic character combination inappropriate for placement in any previously described genus. A new genus, *Rossjoycea*, is proposed for this taxon, so honoring Professor Ross and Mrs. Joyce Bell, the subjects of this Festchrift. Secondly, the New Zealand genus *Meonochilus* Liebherr and Marris (2009)–proposed because its three previously described member taxa did not possess characters consistent with previous classification as *Mecyclothorax* Sharp–is shown to be monophyletic, with three newly described species complementing three species placed there by Liebherr and Marris (2009).

Additionally, this paper serves to establish a starting point for comprehensive morphological analysis of Moriomorphini. As such, discussion of ingroup character transformations is coupled with elucidation of characters that support the dismemberment of Psydrini into an Austral Moriomorphini, and a phylogenetically natural Psydrini; now reduced to three generic taxa; *Psydrus* LeConte, *Nomius* Castelnau, and *Laccocenus* Sloane. The immensely disparate genitalic evolution within Moriomorphini uncovered during character analysis required of the morphologically based cladistic analysis demonstrates that this group will require dense taxonomic sampling in order to fully elucidate the finer points of its evolutionary history.

## Methods

### Taxonomic Material

Taxonomic descriptions in this paper were based on examination of 129 *Meonochilus* and 1 *Rossjoycea* specimen, received on loan from the following institutions: Auckland Museum (AMNZ); The Natural History Museum, London (BMNH); Field Museum of Natural History, Chicago (FMNH); Entomology Research Museum, Lincoln University, Canterbury (LUNZ); New Zealand Arthropod Collection, Landcare Research, Auckland (NZAC); J.I. Townsend personal collection, Levin, New Zealand (JITC), with specimens to be deposited in NZAC. In addition, specimens of other moriomorphine taxa included in the cladistic analysis were borrowed from the Australian National Insect Collection, Canberra (ANIC); Cornell University Insect Collection (CUIC); Museum of Comparative Zoology, Harvard University (MCZ); Naturhistorisches Museum Basel (NHMB); and South Australian Museum, Adelaide (SAM). Australian moriomorphine taxa were identified based on determinations by P.J. Darlington, Jr. (ANIC, MCZ) and Barry Moore (ANIC).

The two species treated as *Moriomorpha* Castelnau in this analysis exhibit bowed mesotibiae, a character used by Moore (1963) to diagnose this genus from *Moriodema* Castelnau. The specimens are identified by locality, as species identification is not currently possible. Moreover, Moore (1963) misidentified *Moriomopha victoriae* Castelnau when he designated this species generotype for *Moriomorpha*. As the holotype specimen of *Moriomorpha victoriae* has straight mesotibiae consistent with Moore’s (1963) diagnosis of *Moriodema* (K. Walker, pers. comm.), and not the bowed mesotibiae used as the diagnostic character for Moore’s concept of *Moriomorpha*, his actions result in synonymy of the names *Moriodema* and *Moriomorpha*, if one assumes mesotibial configuration as the basis for generic recognition. Only a taxonomically more comprehensive cladistic analysis plus taxonomic revision will allow clarification of the taxonomic status of these generic names, and so no nomenclatural conclusions are reached in this matter. Moore’s diagnosis of *Moriomorpha* by the bowed mesotibial configuration is retained for the analysis.

### Laboratory Techniques

Specimen dissection, examination, and photographic protocols follow Liebherr (2009a). Cleared male aedeagal median lobes and parameres, and female reproductive tracts were placed on microscope slides with cover slips prior to photography. When line drawings were prepared, photographs were initially traced to establish the outlines of major structures, and then fine details were added during subsequent examination under phase-contrast compound microscopy. The numbers of individuals used as the basis for the *Meonochilus* genitalic descriptions are provided parenthetically at the start of each genitalic dscription. Dissected genitalic parts were placed in polyethylene vials mounted on the specimen pin, whereas female abdominal ventrites were dried and card-mounted. Moriomorphine specimens used in the cladistic analysis are labeled so that they may be retrieved from the various lending institutional collections (Appendix 1).

When individuals varied in numbers of setae present–e.g., lateral elytral setae, gonocoxal setae, parameral setae–the range of variation is presented when the variation is broadly based. When the vast majority of individuals exhibit one state and only one or several individuals exhibit a second state, the second rare condition parenthetically follows the more common number.

Because *Meonochilus* spp. share many character states and can be diagnosed by only relatively few states, the generic revision first presents a comprehensive generic diagnosis and description relevant to all species. The following species treatments comprise a detailed diagnosis, and separate sections describing the male genitalic and female reproductive tract characters.

Specimen locality data were entered into a flat database, the structure of which is consistent with that described in Liebherr and Zimmerman (2000). Records were grouped by the areas of Crosby et al. (1976). Latitude and longitude were recorded for each locality using the Map Toaster Topo program (Integrated-Mapping.com 2008), Google Maps (Google 2011), or Zenbu (2011). Distribution maps are based on the open-source template available in the *Fauna of New Zealand* series (Manaaki Whenua Press, Lincoln, Canterbury). Holotype labels are presented verbatim, with text on different label lines separated by a slash ( / ), and different labels separated by a double slash ( // ).

### Phylogenetic Analysis

Character descriptions, taxa and character scoring were developed using Winclada (Nixon 2002). The data matrix (Table 2) was first analyzed using Winclada, allowing 200 iterations of the ratchet (Nixon 1999) and other default settings. This result was checked with TNT (Goloboff et al. 2008), using new technology sectorial search, tree fusing, and the ratchet, all at default settings, and finding minimum tree length 200 times.

Characters were treated as either binary, ordered multistate, or unordered multistate (see below). Several character states for several taxa had to be coded as polymorphic due to variation among individuals of the species, and these polymorphisms were incorporated into the Winclada matrix. The TNT search was based on a Nona format file outputted from Winclada, and so the TNT search treated polymorphic terminals as totally ambiguous, not just polymorphic for a subset of possible states. This change did not result in different trees found by TNT versus Winclada.

*Trechus obtusus* Erichson was chosen as the outgroup to which the cladogram of 21 in-group moriomorphine taxa was rooted. This species was chosen because it exhibits generalized external anatomy plus male genitalia of generalized configuration among those taxa phylogenetically placed adjacent to the very diverse carabid radiation classified as Harpalinae (Ober 2002). This generalized condition includes a closed aedeagal median lobe, apically setose parameres of moderate length, and presence of armature on the internal sac (Jeannel 1941). Previously proposed phylogenetic hypotheses that included taxa diverging just prior to the evolution of Harpalinae are various, based both on the types of characters used to derive the hypothesis, as well as on the taxa chosen to investigate different periods of carabid beetle phylogenetic history. Focusing on the more comprehensive molecular phylogenetic analyses, the Moriomorphini have been found to be: 1, the sister group to a clade composed of (Cicindelinae + Rhysodinae + Scaritini + Paussinae), plus Pseudomorphini, Brachinini, and Harpalinae (Maddison et al. 1999); 2, the sister group to Brachini plus Harpalinae (Ober 2002; Ober and Maddison 2008); or 3, sister group to a reduced representation of Harpalinae restricted to *Pterostichus*, *Chlaenius*, and *Galerita* (Maddison et al. 2009). All of these taxa are characterized by male genitalia that plesiomorphically lack the setose parameres and internal sac armature of the Trechitae, reducing the ability to root character transformations within Moriomorphini for these important characters. Choosing *Trechus obtusus* as outgroup serves to provide a stable root for moriomorphine male genitalic characters, as the moderately long setose parameral configuration is broadly distributed across taxa adjacent to Harpalinae. Characters of the moriomorphine male internal sac armature are interpreted in light of the saccal structures elucidated for *Bembidion* by Maddison (1993).

**Table 1.** Key to abbreviations used in illustrations of male genitalia and female reproductive tracts.

Male genitalic structures	Abbreviation	Female reproductive tract	Abbreviation
brush sclerite	b	common oviduct	co
dorsal plate	dp	dorsal ensiform seta	des
flagellum	fl	basal gonocoxite1	gc1
flagellar plate	fp	apical gonocoxite 2	gc2
flagellar sheath	fs	helminthoid sclerite	hs
gonopore	gp	lateroapical seta or setal series	las
left paramere	lp	lateral ensiform setae	les
right paramere	rp	ramus	r
sagittal crest	sc	spermathecal duct	sd
Female reproductive tract	Abbreviation	spermathecal duct callus	sdc
apical nematiform setae	ans	spermathecal gland	sg
bursa copulatrix	bc	spermatheca	sp

**Table 2.** Matrix used as basis for cladistic analysis of Moriomorphini. Characters associated with character numbers presented in text above. Polymorphic characters indicated by bracketed, multiple character states. Inapplicable state indicated by “-.” Unavailable states due to lack of available specimens of particular sex indicated by “?.”

Character number	1 5 10 15 20 25 30 35 40 45 50 55 60 65 70 75
Taxon name	| | | | | | | | | | | | | | | |
*Trechus obtusus*	1000 1 0000000000000100010100000010202000000000 0 [01]0002000100110000000000001200
*Meonochilus amplipennis*	0000 2 0111001030111311011120100100002011101012 0 1 2000010011221130214000112120
*Meonochilus eplicatus*	0000 2 0111001020111311011120100101100011101012[01] 1 1000010011221130213000112120
*Meonochilus placens*	0000 2 0111001010111311011120100101100011101012 1 1 1000010011221130203000112120
*Meonochilus bellorum*	0000 2 0111001020111311001010100100100011100112 1 1 0000010011231110211011112120
*Meonochilus rectus*	0000 2 0111001020111311001120100100100011101012 1 1 0000010011221111213000112120
*Meonochilus spiculatus*	0000 2 0111001020011311001120100100100011101012 1 1 0000010011221111213000112120
*Raphetis darlingtoni*	2100 2 0111000030001310000200101102211111111110 0 1 2000010011221100211012112120
*Selenochilus piceus*	2111 1 1110010021103300100210100222111111111013 0 1 1002011001231100201211110101
*Meonis semistriatus*	2211 1 1110010042102200100102100222212112111112 0 1 1000011001221100111011110011
*Meonis uncinatus*	2211 2 1110010042102200100202000212212112111111 0 1 0000011001221100411123110011
*Mecyclothorax lophoides*	0100 1 0212000010001300001120100000110012100110 1 1 0000010010431110311123122220
*Mecyclothorax montivagus*	0000 1 0212000010001200001110102000100011100112 0 1 0000010010431120004023112220
*Mecyclothorax punctipennis*	0000 1 0212000010001300021111102000100011100112 1 0 0000010010431120004023112220
*Neonomius laevicollis*	0001 1 0111000010103300021010110101100111100110 1 1 0000011101001200201213111020
*Tropopterus duponcheli*	1200[12]02120000220012000000111012023-1011111110 0 1 0000011011221130201013112020
*Amblytelus curtus*	1001 1 0212000020003300020000101000000212100114 0 0 0001011211331100112023112220
*Molopsida pretiosa*	1000 1 0212000031101310001120101102001212100111 1 1 0000010012002210211111111020
*Theprisa australis*	1000 1 0211000030101100011022100102001211111110 0 1 1000010012002200211111111020
*Moriomorpha* sp. “Dunoon, NSW”	2200 1 0211100110202300000002110002001212110111 0 0 01101100?20022003???????????
*Moriomorpha* sp. “Sherbrook, V”	2200 2 0211100110202300000002110002001212100111 0 0 0110110002???????01121111020
*Rossjoycea glacialis*	1000 0 0212000020102000000122100102001211100113 0 1 10000??212???????11111111020

### Phylogenetically Analyzed Characters

The characters below comprised the basis for the cladistic analysis. Multistate characters were treated both as ordered [+], i.e. unit-additive characters, or unordered [-], wherein transformation from one state to any other is assessed one step in the parsimony analysis.

**1.** Labrum apical margin [+]: straight or only broadly and shallowly emarginate (0); emarginate, medial point emarginated 0.1–0.18× apical labrum width (1); very emarginate, medial point emarginated 0.2× labrum width (2). The degree of emargination is measured using a crossed reticle with scale on one axis, rotated 90° to determine first the apical labral width and second the emargination depth.

**2.** Mandibular length [+]: distinctly <2.0× distance from anterior (dorsal) mandibular condyle to labral margin (0); elongate, ~2.0× distance from anterior condyle to labral margin (1); very elongate, >2.0× distance from condyle to labral margin (2).

**3.** Mandibular scrobe ventral margin: extended laterally (0); not extended laterally, the ventrolateral mandibular surface vertical (1).

**4.** Apical maxillary palps [+]: glabrous (0); sparsely covered with short but distinct setae (1).

**5.** Maxillary stipes [+]: with single seta at base (0); with 2 setae near base (1); trisetose, 2 setae near base and 1 seta medially (2). State 2 may be configured with the 2 basal setae in a vertical orientation, or the 2 basal setae may be more horizontally oriented. In either instance the basal setae are separated from the medial seta.

**6.** Lacinial apex: hooked (0); apex rounded, not hooklike (1).

**7.** Glossal sclerite of ligula [+]: narrowly projected, sides parallel (0); narrowed to apex, sides sloping (1); truncate (2). For the two configurations present in Moriomorphini (states 1 and 2) see Liebherr and Marris (2009: Fig. 2, 3).

**8.** Ligular setae across apex of glossal sclerite: 6 (0); 2 (1).

**9.** Paraglossae of ligula [+]: extremely long, thinly extended beyond glossal sclerite, the apical extension much longer than distance from apex of glossal sclerite to paraglossal base (0); moderately long, well extended beyond glossal sclerite anterior margin (1); very short, apex extended just beyond glossal sclerite anterior margin, the apical extension shorter than distance from paraglossal base to apex of glossal sclerite (2).

**10.** Paraglossal articulation with glossal sclerite: adjacent (0); set off laterally from basal portion of glossal sclerite (1).

**11.** Paraglossae of ligula: glabrous (0); setose (1).

**12.** Mentum lateral lobes: extended anteriorly, the mentum deep longitudinally, overall lateral breadth about 2.5–2.8× longitudinal dimension (0); little extended anteriorly, the mentum less deep, overall breadth ≥ 3.0× longitudinal dimension (1).

**13.** Mentum lateral lobes: expanded evenly from anterior apex, not extremely broad (0); broadly expanded laterally from anterior apex, the lateral margin convex (1).

**14.** Mentum medial tooth [-]: bifid (0); broadly rounded (1); narrowly rounded (2); acutely angulate (3); broadly angulate to indistinct (4).

**15.** Mentum paramedial depression [+]: broad, shallowly impressed (0); deep medially but without abrupt pitlike central depression (1); with distinct pit at center of depression (2).

**16.** Antennae [+]: filiform, length of antennomere 9 >2.2× greatest breadth (0); submoniliform, length of antennomere 9 1.5–2.1× greatest breadth (1); moniliform, length of antennomere 9 <1.5× greatest breadth (2).

**17.** Supraorbital setae: 2, both anterior and posterior seta present (0); 1, anterior seta absent (1).

**18.** Frontal grooves [+]: convergent on vertex, extended behind eye (0); sharply impressed, divergent from clypeus to top of eye (1); broadly impressed, divergent from clypeus to top of eye (2); broad and shallow, not linearly impressed (3).

**19.** Vertex microsculpture [+]: evident, distinct isodiametric mesh (0); slightly stretched isodiametric mesh (1); evident to shallow transverse mesh (2); reduced, surface shiny with indistinct transverse mesh (3).

**20.** Pronotal basolateral seta: present (0); absent (1).

**21.** Pronotal lateral seta: near marginal depression or margin (0); distinctly removed from marginal depression, situated on convex disc isolated from depression (1).

**22.** Pronotal lateral seta: near or slightly before pronotal midlength (0); distinctly anterad, near apical 1/4 of pronotal length (1).

**23.** Basolateral margin [+]: distinctly sinuate before obtuse hind angle (0); straight before denticulate hind angle (1); convex, hind angles obsolete (2).

**24.** Pronotal base: impunctate, longitudinal wrinkles may be present (0); punctate (1).

**25.** Pronotal basal margin [+]: with distinct marginal bead present across width (0); without bead medially, distinct raised margin laterally (1); completely unmargined (2).

**26.** Mesepisternum [+]: smooth, without pits (0); punctate, <10 shallow punctures over surface (1); distinctly punctate, >10 deeper punctures present over surface (2).

**27.** Pronotal discal microsculpture [+]: obsolete, surface shiny (0); very shallow to obsolete transverse mesh in parts, surface shiny (1); distinct transverse mesh (2).

**28.** Prosternal process: setose (0); glabrous (1).

**29.** Prosternal process: evenly convex posterad (0); with indistinctly to distinctly keeled posteroventral margin (1).

**30.** Mesothoracic scutellum [+]: broader at base than median length (0); equilateral (1); elongate, basal width < median length (2).

**31.** Parascutellar striole [+]: elongate, evident (0); short, deep or shallow (1); absent (2).

**32.** Elytral basal groove and margin [+]: present, continuous from humerus to parascutellar striole (0); reduced, evident only at humerus (1); absent across base and at humerus (2).

**33.** Elytral humerus [+]: rounded to broadly rounded (0); tightly rounded to subangulate (1); toothed, angulate when basal groove present (2).

**34.** Elytral striae [+]: 1–8 equally impressed, continuous and visible to apex (0); 1–5, 6 or 7 equally impressed, continuous and visible to apex (1); 1–3 impressed on disc (2); only stria 1 impressed on disc, other striae obsolete or interrupted (3).

**35.** Elytral striae: equally developed on disc and at apex (0); reduced at apex, shallower to obsolete (1).

**36.** Elytral striae (where evident) [+]: distinctly punctate (0); minutely or indistinctly punctate (1); smooth (2).

**37.** 7th stria and associated 8th interval [+]: evenly curved to indistinctly convex (0); elevated with tightly rounded apex, subcarinate (1); distinctly and sharply carinate (2).

**38.** Fifth elytral stria: recurrently joining stria to apical margin (0); joining sixth stria and not extended to apical margin (1).

**39.** Subapical sinuation[-]: straight, not evident (0); broadly concave (1); briefly and abruptly concave, plica may be visible from dorsal view (2).

**40.** Elytral internal plica: very narrow, internal, not visible in lateral view (0); deeper and more well developed, visible in lateral view (1).

**41.** Basal elytral dorsal seta: (in “1” position) present (0); absent (1). The numbering system used assumes a ground plan of three dorsal setae, the anterior (or seta 1) near basal quarter to third of length, the apical (or seta 3) well beyond midlength.

**42.** Apical dorsal elytral seta (in “3” position): present (0); absent (1).

**43.** Apical elytral setae: apical seta present only (0; Fig. 11B); both apical and subapical seta (7th stria) present (1; Fig. 11A) (Perrault 1978).

**44.** Elytral lateral setae: 4 in anterior series and 4 in posterior series (0); 6–8 in anterior series and 5–7 in posterior series (1).

**45.** Elytral discal microsculpture (assessed between striae 1–3 in basal half of elytra) [+]: fine transverse lines, iridescent (0); very elongate transverse mesh, iridescent (1); regular elongate transverse mesh (2); isodiametric in transverse rows (3); isodiametric, sculpticells papillate (4).

**46.** Elytral discal microsculpture (mid-intervals): present (0); absent, surface shiny (1).

**47.** Metathoracic flight wings: present (0); vestigial (1).

**48.** Metepisternum-metepimeron juncture [+]: distinct suture, i.e. complete external line from mesal to lateral margins (0); incomplete line, a partial depression on the mesal half of the sclerites (1); indistinct depression, no line (2).

**49.** Mesotibiae: straight (0); bowed convexly outward (1).

**50.** Metatrochanteral apex: rounded (0); attenuated, i.e. pointed (1).

**51.** Tarsomeres dorsal surface [-]: glabrous (0); setose, the setae short (1); setose, the setae long (2).

**52.** Tarsomeres: cylindrical or dorsoventrally compressed (0); bilaterally compressed with excavate lateral areas (1).

**53.** Male ventral protarsal vestiture: uniseriate squamose setae (0); biseriate squamose setae (1).

**54.** Marginal setae, apical visible abdominal ventrite, male: 2 large setae; 2–3 smaller mesal setae also possible (0); 4, 4–5, or 4–6 setae along apical margin (1).

**55.** Marginal setae, apical visible abdominal ventrite, female [-]: 4, 2 each side equidistant from margin (0); 4, 2 each side but medial 2 subapical, set farther from margin than lateral pair (1); 8, 4 setae each side equidistant from margin (2).

**56.** Medial subapical patch of 2–4 short setae, apical visible abdominal ventrite, female: absent (0); present, if four setae present their positions defining a trapezoid with the anterior side the shorter parallel (1).

**57.** Abdominal ventrite lateral depressions (scored on visible abdominal ventrites 3–6) [-]: absent, surface smooth or irregularly wrinkled (0); 1–2 distinct longitudinal depressions (1); 1–2 distinct rounded depressions (2).

**Figure 1.**
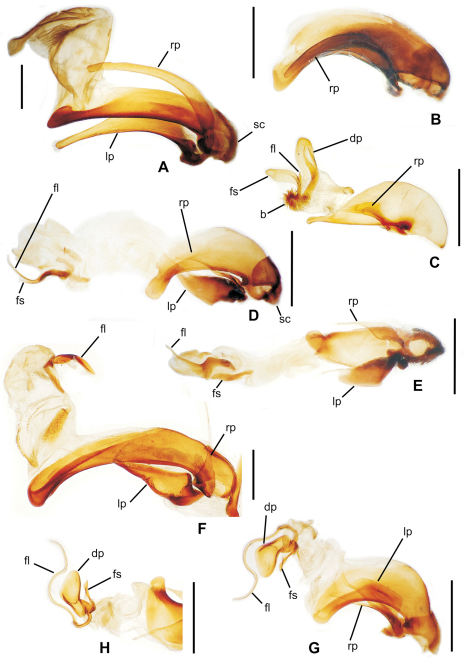
Male aedeagal median lobe and associated parameres, right lateral view, internal sac everted, eudorsal surface toward top of figure (except where noted) **A** *Moriomorpha* sp. “Dunoon, NSW” **B** *Molopsida pretiosa*, internal sac not everted **C** *Trechus obtusus* **D** *Meonochilus bellorum* **E** *Meonochilus bellorum***,** ventral view **F** *Amblytelus curtus* **G** *Mecyclothorax lophoides* **H** *Meonochilus lophoides*, left lateral view (see Table 1 for abbreviations; scale bars = 0.5 mm).

**58.** Ventral (i.e. right except for *Tropopterus* Solier, which has the aedeagus inverted) paramere shape [+]: elongate styloid, apex broadly subparallel from basal quarter to rounded tip (0; Fig. 1A, 1B); conchoid, basal portion distinctly broader than apical quarter, apex rounded (1; 1C); elongate conchoid, apex narrowed (2; Fig. 1D, 1E); elongate, styloid, apex narrowed to whiplike extension (3; Fig. 1F); more elongate whip, paramere >0.85 median lobe gape (4; Fig. 1G). The gape of the median lobe is defined as the distance from the bosslike sclerotized point of articulation of the parameres, to the convex external face of the lobe apex.

**Figure 2.**
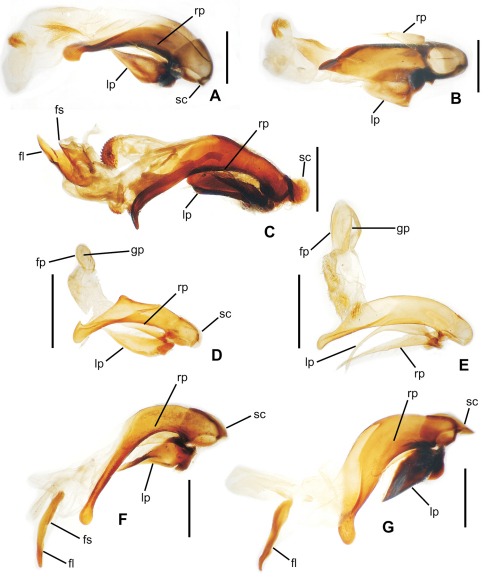
Male aedeagal median lobe and associated parameres, right lateral view, internal sac everted, eudorsal surface toward top of figure (except where noted) **A** *Meonochilus amplipennis***B** *Meonochilus amplipennis*, ventral view **C** *Meonis uncinatus* **D** *Mecyclothorax dentatus* **E** *Mecyclothorax punctipennis* **F** *Meonochilus spiculatus* **G** *Meonochilus rectus* (see Table 1 for abbreviations; scale bars = 0.5 mm).

**59.** Dorsal (i.e. left except for. *Tropopterus*) paramere shape [+]: broader basally, apical half elongate, subparallel (0; Fig. 1A); sides sinuously subparallel, apex short and broad (1; Fig. 1C); broad basally, apex short, narrowly rounded, subangulate (2; Fig. 2A, 2B); broader basally, apex elongate and much narrower, attenuate (3; Fig. 1G).

**60.** Ventral (right except for *Tropopterus*) paramere setation [+]: large setae only at apex (0; Fig. 1C); setose on ventral, apical and/or dorsal surfaces, setae evident (1; Fig. 1G); setae present but very small, or paramere glabrous (2; Fig. 1A).

**61.** Dorsal (left except for *Tropopterus*) paramere setation [+]: 5 large setae at apex (0; Fig. 1C); 2–4 larger setae at apex, additional smaller setae may be present (1; Fig. 10); from 1–6 very short setae at apex, or apex glabrous (2; Fig. 1A).

**62.** Male aedeagal internal sac flagellum [-]: short pointed structure associated with central sclerite complex (CSC, Maddison 1993: 167) (0; Fig. 2C); elongate whiplike structure associated with CSC (1; Fig. 1G, 1H); median scoop-like plate supporting gonopore on dorsum (2; Fig. 2D, 2E); absent (3; Fig. 2A, 2B). Assignment of the flagellum to the central sclerite complex follows the terminology established by Maddison (1993) for *Bembidion*. The scooplike sclerite of the internal sac observed in most *Mecyclothorax* has been interpreted previously as a dorsal plate (e.g., Liebherr 2009a; Liebherr and Marris 2009). This scooped lobe at the apex of the internal sac comprises a concave, sclerotized ventral surface, often bearing radially oriented, thicker sclerotized ridges, and a membranous dorsal surface bearing a medially positioned gonopore (e.g., Fig. 2D – *Meonochilus dentatus* Liebherr of Oahu). In several undissected *Mecyclothorax* individuals, spermatophores have been observed projecting from this dorsomedial gonopore (unpubl. data), verifying the gonopore’s position. As the dorsal plate of *Bembidion* lies dorsad the gonopore and has a sclerotized dorsal surface, and the *Bembidion* flagellar structures are more closely associated with the gonopore (Maddison 1993, also presently observed in *Bembidion (Peryphus) transversale* (Djean) and *Bembidion (Zeplataphuscharile* Bates), the sclerotized scoop-like ventral surface observed in *Mecyclothorax* is not positionally homologous with the dorsal plate of *Bembidion*. In order for the previously named *Mecyclothorax* “dorsal plate” to be homologous with that observed in *Bembidion*, an evolutionary transformation of gonopore position from ventrad the dorsal plate (in *Bembidion*), to the dorsal surface of the dorsal plate (in *Mecyclothorax*) must be hypothesized. Conversely, giving primacy to the position of the gonopore, the sclerotized scoop-like structure at the apex of the *Mecyclothorax* internal sac may be reinterpreted as a modification of the flagellum with associated evolutionary loss of the original dorsal plate. To date, no moriomorphine taxa have been observed to possess both an elongate flagellum and a scoop-like sclerite ventrad the gonopore. A more thorough sampling of taxa throughout the Trechitae and associated groups is required to assess these competing hypotheses regarding the homology of the scoop-like sclerite. In this study the term flagellar plate replaces the previously used term dorsal plate (e.g., Liebherr 2007, 2011) to describe the sclerotized scoop-like structure on the *Mecyclothorax* sac. The unordered treatment of this character is compatible with this assessment of flagellar homology, as it leaves ambiguous any hierarchical relationship of the various flagellar configurations.

**63.** Flagellum configuration: absent, short, thin, or whiplike, flagellar sheath independent (Maddison 1993) (0; Fig. 1D, 1G, 2C); stout, spikelike, flagellar sheath tightly appressed to flagellum (1; Fig. 2F, 2G). The flagellar sheath in Moriomorphini is interpreted as the shorter structure, appressed (Fig. 1E) or not (Fig. 2C), that is associated with a longer flagellum. The moriomorphine flagellar sheath lies to the left of the longer flagellum in *Meonochilus bellorum*, sp. n. (Fig. 1E) and *Meonochilus spiculatus*, sp. n. (Fig. 2F), and dorsad the flagellum in *Meonis uncinatus* Baehr (Fig. 2C). Thus even within Moriomorphini the position of these respective structures is labile. The functional terminology of flagellum and flagellar sheath adopted here must be tested using a broad array of Trechitae and associated Moriomorphini in order to establish reliable homology statements relating the two structures. However, the derived state of this particular character focuses on the spikelike flagellar configuration observed only within *Meonochilus* *spiculatus* and *Meonochilus rectus*, sp. n., ensuring this character’s appropriate inclusion in the cladistic analysis.

**64.** Aedeagal median lobe tip [-]: extended narrowly beyond ostium (0; Fig. 2E); narrow, not extended beyond ostium (1; Fig. 1F); broadly expanded dorsoventrally, extended beyond ostium (2; Fig. 2A); broadly expanded dorsoventrally, not extended beyond ostium (3; Fig. 1G); expanded laterally, extended beyond ostium (4; Fig. 2C).

**Figure 3.**
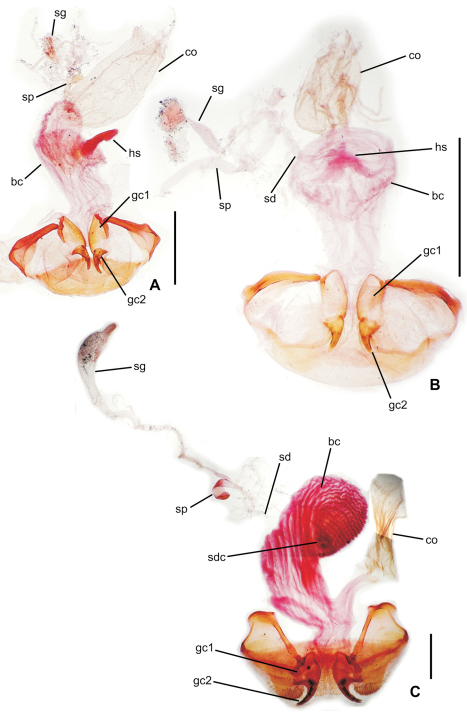
Female reproductive tract, ventral view **A** *Selenochilus piceus* **B** *Molopsida pretiosa* **C** *Amblytelus curtus* (see Table 1 for abbreviations; scale bars = 0.5 mm).

**65.** Female bursa copulatrix shape: elongate columnar, length/width >2.0–3.25 (0; Fig. 3A); columnar, length/width 1.0–2.0 (1; Fig. 3B).

**Figure 4.**
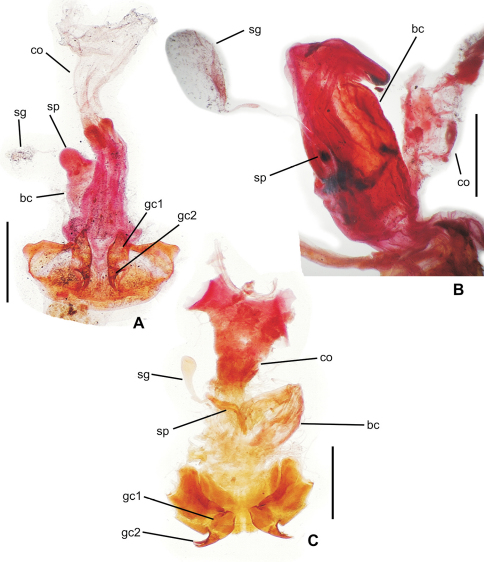
Female reproductive tract **A** *Meonochilus spiculatus*, ventral view **B** *Meonochilus amplipennis*, left dorsolateral view**C** *Raphetis darlingtoni*, ventral view (see Table 1 for abbreviations; scale bars = 0.5 mm).

**66.** Spermathecal placement [-]: on common oviduct (0); ventrally on common oviduct/bursa juncture (1; Fig. 3B); ventrally on bursa copulatrix (2; Fig. 3C); apically on bursa copulatrix (3; Fig. 4A); dorsally on bursa copulatrix (4; Fig. 4B).

**67.** Helminthoid sclerite at base of spermathecal duct [-]: absent (0; Fig. 4A); comprising sclerotized, roundly projected base of spermathecal duct (1; Fig. 3B); consisting of elongate sclerotized apodeme (2; Fig. 3A).

**68.** Spermathecal duct length [+]: zero, spermatheca appressed to wall of bursa copulatrix (0; Fig. 4A, 4B); subequal to spermathecal reservoir length (1; Fig. 3B); 1.5–3.0× spermathecal reservoir length (2; Fig. 3C).

**69.** Spermathecal shape [-]: appressed bulb(s) (0; Fig. 4B); unsclerotized tube subequal in diameter to spermathecal duct (1; Fig. 3A); sinuous sclerotized tube subequal in diameter to spermathecal duct (2; Fig. 4C); ovate or obovate reservoir broader than spermathecal duct (3; Fig. 3C).

**70.** Spermathecal gland: absent (0); present and entering base of spermathecal reservoir (1).

**71.** Ramus (gonopod VIII of Deuve 1993) [+]: absent (0); present and membranous (1); present, sclerotized (2).

**Figure 5.**
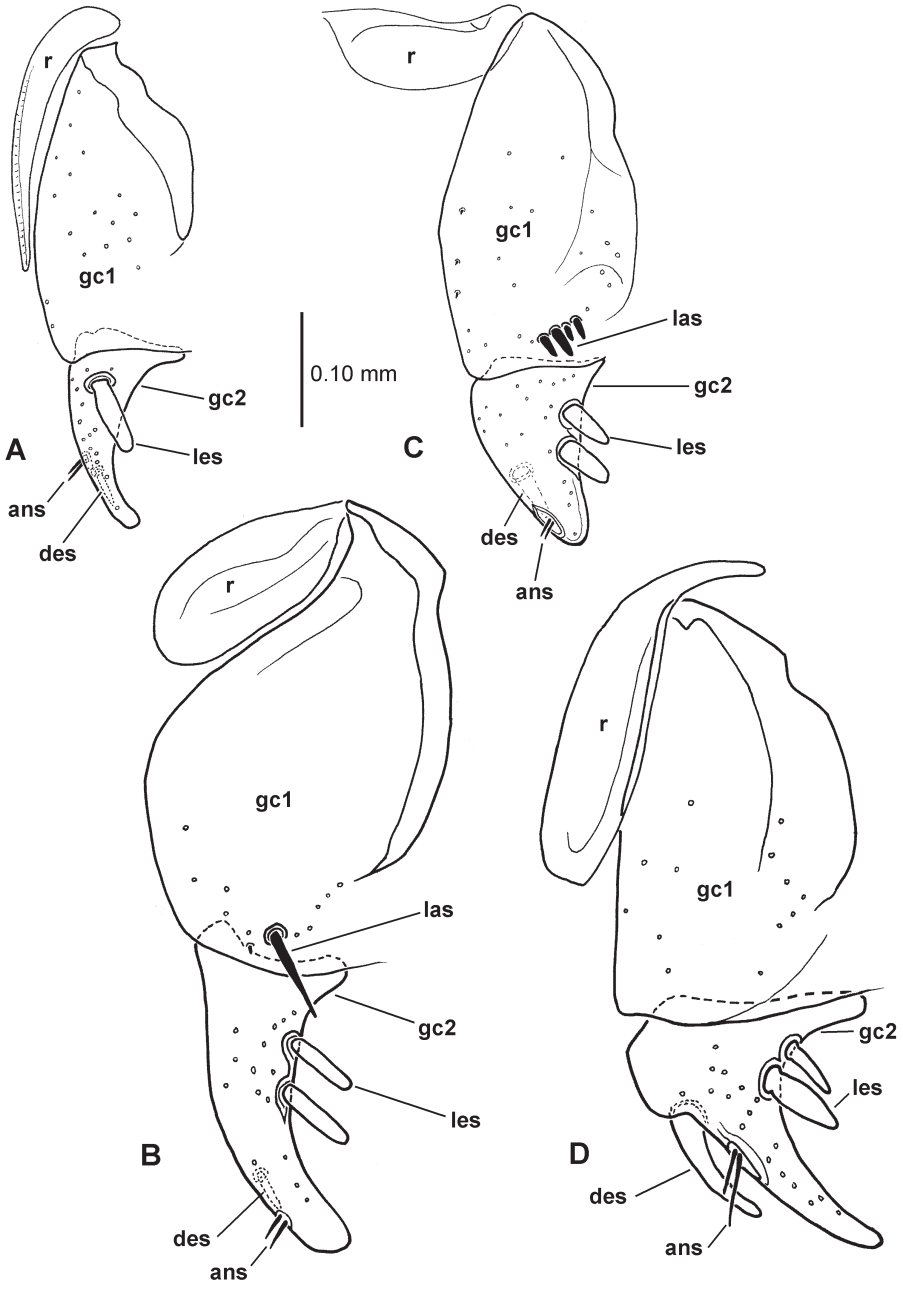
Female left gonocoxite, ventral view **A** *Selenochilus piceus* **B** *Moriomorpha* sp. “Sherbrook, V” **C** *Meonochilus amplipennis* **D** *Meonis uncinatus* (see Table 1 for abbreviations).

**72.** Lateroapical fringe of setae on gonocoxite 1 [+]: absent (0; Fig. 5A); composed of single seta (1; Fig. 5B); composed of 2–5 setae (2; Fig. 5C).

**73.** Medial setae of gonocoxite 1 [-]: absent along entire mesal margin (or only a single small seta) (0); all setae similarly small, setae arrayed along mesal surface (1); larger seta at apical angle plus other setae along mesal margin (2).

**74.** Lateral ensiform setae of gonocoxite 2 [-]: only 1 seta (0); 1 larger seta plus 1 small seta (1; Fig. 5D); 2 similarly sized setae (2; Fig. 5C).

**75.** Apical sensory furrow: near apex of gonocoxite 2, position 0.70–0.88 of gc2 length (0; Fig. 5C); more medial, position 0.55–0.60 of gonocoxite 2 length (1; Fig. 5D).

**Figure 6.**
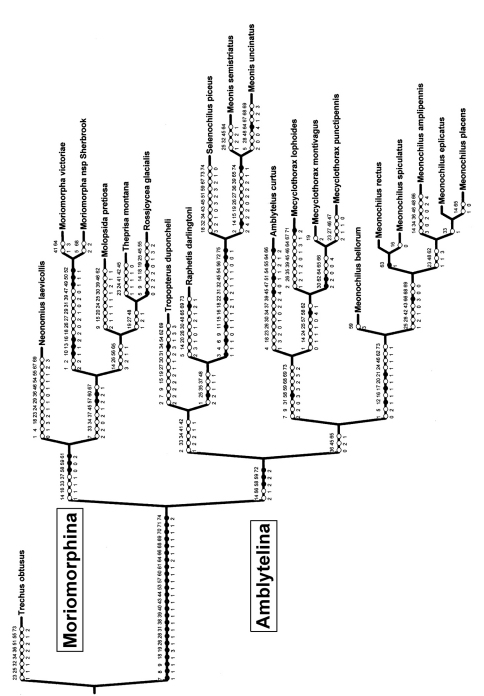
Single most parsimonious, 301-step cladogram of ingroup Moriomorphini rooted at *Trechus obtusus* outgroup, showing character state transformations along edges of cladogram; character numbers above edges, states at that edge shown below.

## Results of Phylogenetic Analysis

Parsimony analysis using both the Winclada and TNT programs resulted in a single tree (Fig. 6) of length 301 (CI = 0.43, RI = 0.63). The moriomorphine taxa included in the analysis are divided into two clades defined by male aedeagal configuration. The clade subtended by *Neonomius laevicollis* (Sloane) is characterized by male parameres that are elongate, with the ventral and dorsal margins parallel for much of their length (character 58, state 0). The sister group–*Tropopterus* to *Meonochilus* clade (Fig. 6)–exhibits a ground-plan of parameres that are moderately elongate, narrowed apically and thus of conchoid shape, but without narrow apical whip-like extensions (character 58, state 2). From this configuration subsequently arises the elongate, apically narrowed, styloid to whip-like parameres observed in species of *Amblytelus* Erichson and *Mecyclothorax* (character 58, states 3 and 4). Parameral setation is congruent and concordant with this dichotomy, with the elongate, paddle-like–i.e. parallel-sided–parameres of the *Neonomius* Moore clade being glabrous or bearing only very short setae (character 61, state 2).

The setation of the female gonocoxae also supports this dichotomy, with the *Neonomius* clade retaining the symplesiomorphic condition of a single seta comprising the lateroapical series of seta on basal gonocoxite 1, whereas the *Tropopterus*-*Meonochilus* clade is characterized by a ground-plan state of two or more setae in this series. Within this clade, the genera *Selenochilus* Chaudoir and *Meonis* Castelnau deviate by exhibiting basal gonocoxites that lack the lateroapical setal series; i.e. they are glabrous.

The presence of a basally sclerotized spermathecal duct, or helminthoid sclerite–the various configurations of which were shown to be independently derived–also defines the *Neonomius* clade. Whereas *Neonomius* *laevicollis* exhibits an elongate sclerotic apodeme at the spermathecal duct base (character 67, state 2), *Moriomorpha*, *Molopsida* White, *Theprisa* Moore, and *Rossjoycea* have the spermathecal duct base sclerotized as a rounded projection (state 1). These two configurations recur in other taxa; the state 2 sclerotic apodeme in *Selenochilus*, and the state 1 rounded spermathecal duct base in *Meonis uncinatus* and *Mecyclothorax lophoides* (Chaudoir).

Other characters define less inclusive clades, or support groupings inconsistent with the overall division supported by the male parameral configuration. These are treated successively by groupings of characters derived from the mouthparts, elytra and metathoracic flight wings, abdominal ventrites, male genitalia, and female reproductive tract.

### Mouthparts

Thomas Broun utilized the configuration of the labrum extensively in his taxonomic work (e.g., Broun 1912: 387). The degree of emargination of the labral anterior margin varies significantly across Moriomorphini, with *Moriomorpha*, and the clade comprising *Raphetis* Moore, *Selenochilus* and *Meonis* both exhibiting a labrum with the median point on the margin emarginated more than 0.2× the labral width (character 1, state 2). These independent derivations of an emarginated labrum are mirrored in mandibular configuration, with both *Moriomorpha* and *Meonis* characterized by very elongate mandibles (character 2, state 2), and *Raphetis* and *Selenochilus* possessing elongate mandibles (state 1). For two of these taxa–*Selenochilus* and *Meonis*–these first two conditions are joined by a number of other restricted synapomorphies, resulting in a mouthpart syndrome that is unique within Moriomorphini. These synapomorphies include: 1, the mandibular scrobe lacking a horizontal ventral margin, with the outer face of the mandible principally vertical (character 3, state 1); 2, the apical maxillary palpomere sparsely covered with short but distinct setae (character 4, state 1), though this is also observed in *Amblytelus*; 3, the maxillary lacinia with a rounded apex, unlike the generalized hooked, acuminate apex in all other taxa (character 6, state 1); 4, setose paraglossae, their surfaces densely covered with felt-like protrusions (character 11, state 1). These mouthpart characters suggest similar feeding habits for *Selenochilus* and *Meonis* beetles, with two other characters of the forebody adding to this conjecture; 1, the lateral pronotal setae are positioned in the anterior ¼ of the pronotum (character 22, state 1); and 2, the basal groove and margin of the elytra is variously reduced or absent (character 32, states 2 and 3). All of these characters would be consistent with these beetles entering narrow voids to feed, during which they would use their narrow, acuminate mandibles to slash their prey. Baehr’s (2007: 569) hypothesis that their “cychroid body shape with smooth and narrow fore body and very elongate, pincer-like mandibles suggests either a diet of shelled snails or a method of picking insects, worms, etc. from deep cracks in the bark or trunks of logs” is a good starting point for predicting their prey preferences. Regarding their phylogenetic relationships, the evidence from this functional suite of characters is not countermanded by other characters, and so it would appear that *Selenochilus* and *Meonis* are sister genera, there being no other similar taxa not included in the present cladistic analysis.

The apical margin of the glossal sclerite has been used to differentiate taxa within this assemblage (Moore 1963; Liebherr and Marris 2009). Based on the cladistic hypothesis and an ordered transformation series rooted at Trechini (with a narrow, parallel-sided glossal sclerite), the plesiomorphic condition of the glossal sclerite within Moriomorphini is one that has a narrowly rounded apical margin, with this shape shared by *Neonomius*, the *Raphetis-Meonis* clade, and *Meonochilus* (character 7, state 1). An anteriorly truncate glossal sclerite (state 2) is thus hypothesized to have evolved independently in the *Moriomorpha-Rossjoyeca* clade, in *Tropopterus*, and in the *Amblytelus-Mecyclothorax* clade. However this solution is strictly based on the chosen outgroup and the constellation of ingroup taxa. This evolutionary scenario needs to be tested in a more comprehensive analysis.

### Elytra and metathoracic flight wings

The plesiomorphic configuration of the parascutellar striole in Moriomorphini is a foreshortened striole that may be deep or shallow (character 31, state 1). This configuration may evolve to complete reduction of the striole, observed in *Tropopterus*, and the *Selenochilus-Meonis* generic pair, or the striole may be elongate as in the generalized carabid condition; this state (0) observed in those lineages including generic taxa with at least some species exhibiting fully developed metathoracic flight wings (character 47, state 0). Based on the cladistic analysis and included taxa, one might be tempted to optimize wing evolution so that wing loss was reversed from the vestigial condition, with fully developed wings in *Moriomorpha*, *Amblytelus*, and *Mecyclothorax punctipennis* being secondarily derived, as hypothesized by Whiting et al. (2003) for stick insects. However the evolutionary ease with which flight wings are reduced in Carabidae, in association with occupation of stable, persistent habitats (Darlington 1943; Southwood 1977) means that a hypothesis of recurrent wing evolution must factor in the probability of loss in the first instance. For the current cladogram, strict interpretation of wing recurrence from the vestigial state entails three evolutionary reversals from vestigial wings to the fully winged state, whereas assumption of recurrent, independent wing loss requires six transformations. This disparity is judged insufficient to support an interpretation of wing recurrence given the repeated loss of wings in numerous distantly related carabid lineages (Darlington 1943). In any event, this character contributes little to the cladistic structure of the hypothesis.

The presence of a tooth on the elytral margin at the angulate juncture of the lateral and basal grooves–i.e. on the humerus–provides grouping information for two clades. These include the *Moriomorpha-Rossjoycea* clade, and the *Tropopterus-Meonis* clade. In the former instance, the toothed margin has been derived from an untoothed, angulate humerus as observed in *Neonomius*. The presence of a subapical carina on the inner margin of the eighth elytral interval, just laterad the seventh stria, also groups taxa in the *Moriomorpha-Rossjoycea* clade, though it also independently characterizes *Amblytelus*. Such a raised eighth interval also has evolved independently in *Mecyclothorax*, being observed in Tahitian species of the *Meonochilus altiusculus*, *Meonochilus globosus*, *Meonochilus gourvesi*, and *Meonochilus striatopunctatus* species groups (Perrault 1986, 1988). Thus this structure has evolved independently a number of times, and its use in classification should be restricted to situations where its independent derivation is congruent with other characters.

### Abdominal ventrites

The abdominal ventrites of many Moriomorphini exhibit longitudinally wrinkled lateral surfaces, with one or two of these wrinkles being deeper and longer. Based on the cladistic hypothesis, this condition comprises part of the ground plan for the tribe (character 57, state 1). These deeper wrinkles are not observed in *Mecyclothorax* spp., where the lateral reaches of the ventrite may be irregularly wrinkled, or variously wrinkled in different individuals. Conversely, the lateral reaches of the ventrite surface bear rounded depressions in the *Moriomorpha-Rossjoycea* clade. Whether these deeper wrinkles and rounded depressions represent external evidence of internal muscle attachments developing in the pupal stage will require internal dissection of the abdominal muscle system.

### Male genitalia

The moriomorphine ground plan includes presence of a short flagellum associated with the central sclerite complex on the male aedeagal internal sac, similar to that described by Maddison (1993) for *Bembidion*. Such a flagellum is also observed in Trechini (Fig. 1C). The flagellum has taken on an elongate configuration independently several times among the taxa studied here; in *Molopsida*, *Mecyclothorax lophoides*, and in *Meonochilus bellorum*, *Meonochilus rectus*, and *Meonochilus spiculatus* (Fig. 1B, 1D, 1G, 2F, 2G). Within beetles currently classified as *Mecyclothorax*, a second flagellar configuration–the flagellar plate (Fig. 2E) – has evolved. The flagellar plate bearing *Mecyclothorax* are present in Australia, and also comprise the entirety of the various non-Australian radiations; e.g., those in New Guinea, New Caledonia, New Zealand, the Society Islands, and Hawaii. Further analysis of the internal sac configurations among the Australian species will establish the cladistic structure from which the various island radiations have sprung.

### Female reproductive tract

Liebherr and Will (1998) classified *Psydrus* and various moriomorphine taxa together in an obsolete concept of Psydrini, however variation among the presently studied moriomorphine taxa demonstates that evolution of the female reproductive tract system is much more complicated than previously reported. In particular, the position and configuration of the female spermatheca varies dramatically. In the ground-plan condition, the female spermatheca enters the reproductive system via a duct that is joined near the juncture of the common oviduct and the bursa (Fig. 3B). This spermathecal placement represents the generalized condition of Carabidae (Liebherr and Will 1998) that is consistently observed in all genera of this analysis save *Amblytelus*, *Mecyclothorax* and *Meonochilus*. In the former, based on *Amblytelus curtus* (F.), the spermathecal duct joins the bursa at a dome-like structure appressed to the ventral surface of the bursa, some distance apicad the common oviduct-bursal juncture (Fig. 3C). The vast majority of species of *Mecyclothorax* for which the female tract has been described (Liebherr 2005, 2007, 2008a, b, 2009a, b, 2011; Liebherr and Marris 2009) have an elongate spermathecal duct that is joined to the dorsal surface of the bursa (as shown in Liebherr and Will 1998). But, this is not seen in *Meonochilus lophoides*, in which the spermatheca is in the plesiomorphic position, joined by a duct to the common oviduct-bursal juncture. Within *Meonochilus*, the spermathecal position varies even more dramatically, with *Meonochilus bellorum* exhibiting the plesiomorphic condition (Fig. 12A), *Meonochilus spiculatus*, *Meonochilus rectus*, *Meonochilus eplicatus* (Broun), and *Meonochilus placens* (Broun) having an appressed, domelike or very indistinct spermatheca entering near the apex of the bursa (Fig. 4A, 14B, 17A, 17C), and *Meonochilus amplipennis* (Broun) having the spermatheca comprising a domelike structure on the dorsal surface of the bursa (Fig. 4B). Reduction of the spermatheca from an elongate reservoir on a duct to an appressed button on the bursal wall has occurred independently in the Platynini; subgenus *Batenus* Motschulsky of *Platynus* Bonelli (Liebherr 1989).

Deuve (1993) hypothesized that the lobate sclerite or membranous folds mesad the base of the female gonocoxae IX represent gonopods of abdominal segment VIII. Liebherr and Will (1998) interpreted these structures as secondarily derived, using the neutral term ramus (Bils 1976) for them, and coded the psydrine subtribes as lacking them. Based on the presence of well-sclerotized folds of membrane between the gonocoxites of female *Mecyclothorax lophoides*, it is apparent that the membranous lunules reported for other *Mecyclothorax* spp. (e.g., Liebherr 2007) should be interpreted as membranous rami. Learning the full distribution of this character across the various tribes annectant to the Broscitae and Harpalinae will determine how this character should be interpreted (Johns 2010).

## Classification of Moriomorphini

### Monophyly of Moriomorphini

Cladistic support for Moriomorphini relative to the *Trechus* *obtusus* outgroup is abundant, with unreversed synapomorphies relative to *Trechus* including: 1, bisetose glossal sclerite (character 8); 2, male protarsal adhesive setae biseriate; 3, male ventral paramere with ventral margin setose, or parameral setae reduced in length so the paramere appears glabrous; 4, male dorsal paramere with only 2–4 apical setae, or from 1–6 very short apical setae; 5, appended female spermathecal gland present. More importantly for substantiating monophyly of the relatively newly recognized assemblage of genera included in Moriomorphini is the placement of other genera previously included in a more inclusive Psydrinae, or Psydrini (Moore 1963; Baehr 1998). These genera include the Australian *Laccocenus* Sloane, the North American *Psydrus* LeConte, and the Holarctic *Nomius* Castelnau. These three genera were considered to represent the “most primitive tribe in the subfamily” Psydrinae by Moore (1963), suggesting he viewed them as the aggregate sister group to the rest of his Psydrinae. Baehr (1998) placed Psydrini as outgroup to a clade comprising Patrobinae plus the Austral subtribes that are treated herein as Moriomorphini. Symplesiomorphies of the Psydrini included: 1, dorsally setose tarsomeres; 2, antennae setose from the basal antennomere, 3, male ventral protarsal vestiture spongiose, not biseriate; and 4, parameres large and of similar shape. Synapomorphies supporting an Austral clade included: 1, basal bulb of male aedeagal median lobe closed; 2, antennae setose from antennomere 4; 3, tooth of mentum unidentate.

The Psydrini sensu stricto were also found to differ substantially from the Austral groups herein considered Moriomorphini in a cladistic analysis including Broscinae, Harpalinae, and Trechitae (Roig-Juñent and Cicchino 2001).The Austral groups–Meonini, Mecyclothoracini, Tropopterini, Melisoderini and Amblytelini–did not constitute a monophyletic group in their analysis, being paraphyletically arrayed between the broscines, harpalines and trechines. However several of the character states dividing the Austral groups were incorrectly coded in their analysis: 1, Meonini was incorrectly scored as lacking an appended spermathecal gland; 2, the left paramere of Amblytelina was incorrectly coded as glabrous; 3, only Amblytelina was coded for presence of a ramus, though more thorough study suggests all moriomorphines have at least a membranous ramus; and 4, Mecyclothoracini was coded for absence of dorsal elytral setae. The character states associated with parameral setation reverse within the network of Austral groups. Moreover, they differentially coded the attachment of the ducts of the pygidial defense glands, with some Austral taxa coded for gland ducts entering on the posterior margin of tergite VIII, and some taxa coded for glands entering on the anterior margin of tergite IX (states described in Deuve 1993). Careful examination of the gland duct attachments on all moriomorphine taxa included in this analysis showed that the ducts attach to the membrane between tergites VIII and IX, consistent with the point of attachment observed in “Psydridae and Trechidae” (Deuve 1993: 44, Fig. 16). Finally, they coded all “psydrine” taxa–Psydrini plus the various Austral groups–as possessing a grade B antennal cleaner (Hlavac 1971). The mesal groove of the antennal cleaner in *Psydrus*, *Nomius* and *Laccocenus* extends well above the clip setae, adhering to the grade B configuration; as reported for the former two genera by Hlavac (1971: Table 1). However the moriomorphine genera have a much shorter mesal groove that barely extends dorsad the clip setae and the setal band is more clearly divided into a long, straight distal region, and a curved proximal cleaning section, consistent with assignment to the more derived and synapomorphous antennal cleaner configuration categorized by Hlavac as “grade C.”

Finally, ribosomal 18S molecular sequence data (Maddison et al. 1999) grouped several Austral groups herein considered Moriomorphini, placing *Laccocenus* far removed in association with Broscini. This result has been corroborated using combined information from 18S and 28S ribosomal DNA, and the wingless gene, monophyletically grouping *Meonis*, *Melisodera* Westwood, *Sitaphe* Moore, *Tropopterus*, *Amblytelus* and *Mecyclothorax* relative to a broad array other carabid taxa (D.R. Maddison and K. Ober, pers. comm.). Thus repeated results support: 1, the “true” Psydrini–*Psydrus*, *Nomius*, *Laccocenus*–are not most closely related to taxa herein classified as Moriomorphini; and 2, the Moriomorphini represent an Austral disjunct monophylum spanning the continents of Australia including New Guinea, New Zealand, and South America, as well as various island groups in the Indian and Pacific Oceans.

### Subtribal classification

Based on the character distributions of the cladistic hypothesis, principally those of the male parameres, a two-subtribe classification is herein proposed. The nominate subtribe Moriomorphina (Fig. 6) includes those taxa with elongate, parallel-sided parameres that are glabrous or only sparsely clothed with very short setae. These generic taxa include *Neonomius* Moore, *Moriomorpha* Castelnau, *Molopsida* White, *Theprisa* Moore and *Rossjoycea*, gen. n. of the present analysis. Based on Moore (1963), other Australian genera that he placed in the Melisoderini and “Tropidopterini” (Tropopterini) also should be assigned to Moriomorphina: *Celanida* Castelnau, *Melisodera* Westwood, *Moriodema* Castelnau, *Pterogmus* Sloane, *Rhaebolestes* Sloane, *Sitaphe* Moore, *Teraphis* Castelnau, and *Trephisa* Moore. The sister group, Amblytelina, includes those taxa with more setose parameres, either of the conchoid shape wherein the parameres are shorter, basally broader and narrowly rounded apically. It also includes those taxa with elongate setose parameres that bear a variably extended, whiplike apex. Recognition of the latter as distinct (such as the previous interpretations of Amblytelina and Mecyclothoracina; Moore 1963), requires those taxa with setose conchoid parameres to comprise a paraphyletic taxon. Genera assigned to Amblytelina included in this analysis are: *Amblytelus* Erichson, *Mecyclothorax* Sharp, *Meonis* Castelnau, *Meonochilus* Liebherr and Marris, *Raphetis* Moore, *Selenochilus* Chaudoir, and *Tropopterus* Solier. Additional related taxa that should also be assigned include *Epelyx* Blackburn, *Districhothorax* Blackburn, *Paratrichothorax* Baehr, *Pseudamblytelus* Baehr, and *Trichamblytelus* Baehr (Baehr 2004).

The results of this cladistic analysis necessitate major classificatory transfers of several of the above genera. *Neonomius* was considered the sister genus to *Mecyclothorax* by Moore (1963), consistent with the included species first being described in that genus, and the microsympatic cooccurrence of individuals representing both genera in Australian mesic *Eucalyptus* forest habitats (unpubl. data). However, parameral shape and setation (characters 58, 59, 61) point directly to membership in Moriomorphina (Fig. 6).

The name Tropopterini, first proposed as Tropopterides by Sloane (1898), has been consistently misapplied to Australian and New Zealand taxa throughout the history of the name, without any discussion of underlying characters that support such a decision. The male parameres of the South American *Tropopterus* *duponcheli* Solier are expanded basally, with the left paramere abundantly setose on the ventral margin, and the right bearing a setose apical extension from a broader base. These configurations are reversed from the rest of the Amblytelina because the male aedeagus rests on its left side in repose; i.e. it is “inverted.” Broun (1880) initially described three New Zealand species in *Tropopterus*; the currently combined *Molopsida sulcicollis*, *Molopsida seriatoporus*, and *Meonochilus placens*. All three species were recombined with *Tarastethus* by Sharp (1886). Independently, White’s (1846) genus name *Molopsida*, treated as a pterostichine (Csiki 1930), was assigned as the senior synonym of *Tarastethus*, linking the name *Molopsida* to New Zealand taxa originally described in *Tropopterus*, with both names assigned to the Nomiini (= Psydrini). Thus without any direct comparison of specimens in the respective genera, the names *Tropopterus* and *Molopsida* became nomenclaturally linked, an ironic twist given that Sloane’s (1898) original generic roster of Tropopterides mentioned only *Tropopterus* and *Cyclothorax* Blackburn (= *Mecyclothorax*). The present cladistic analysis clearly demonstrates that the South American *Tropopterus* belongs to a clade also including the Australian genera *Raphetis* and *Meonis*, plus New Zealand’s *Selenochilus* (Fig. 6). Conversely, *Molopsida* is a distant relative in Moriomorphina, placed near *Theprisa* from Australia and *Rossjoycea* from New Zealand.

### Monophyly of *Meonochilus*

The monophyly of *Meonochilus*, necessary for a stable revision of the species, is supported by several characters. Most prominently, the head of *Meonochilus* bears only the posterior of the two plesiomorphically present supraorbital setae. The anterior supraorbital seta is lost in more than several *Mecylothorax* (e.g., Liebherr 2008a), necessitating other characters to support the diagnosis. This support is provided by the pronotal setation in *Meonochilus*, wherein the basal pronotal seta, plesiomorphically present at the hind angle, is absent, and the lateral pronotal seta is isolated from the marginal depression, emanating from the lateral portion of the pronotal disc. Finally, the mentum is foreshortened longitudinally relative to its breadth (character 12, state 1).

## Zoogeography of New Zealand Moriomorphini

The New Zealand moriomorphine fauna comprises taxa placed in *Molopsida*, *Rossjoycea*, *Selenochilus*, *Mecyclothorax*, and *Meonochilus* (Fig. 6). In all instances, these New Zealand representatives are most closely related to Australian taxa. The sole South American moriomorphine–*Tropopterus*–subtends a clade including taxa from Australia and New Zealand, suggesting that Cretaceous to Early Oligocene-aged vicariance of gondwanan areas, if it influenced diversification of Moriomorphini, occurred prior to the diversification of the various taxa from Australia and New Zealand. The relationships of the various Australian-New Zealand moriomorphine taxa are more likely associated with the subsequent, episodic zoogeographic connections of Australia and New Zealand via the Reinga-Norfolk Ridge system (Herzer et al. 1997; Chambers et al. 2001).

The geographic restriction of *Meonochilus* to the North Island of New Zealand corroborates the importance of a northerly zoogeographic connection between Australia and New Zealand during the evolution of this group. Moreover, *Meonochilus* species diversity is highest on the isolated mountains and islands of Northland, Auckland, the Coromandel Peninsula, and Bay of Plenty, with only *Meonochilus amplipennis* distributed over the extensive, younger portions of the island south of Bay of Plenty (Fig. 13, 15). This pattern of highest diversity in areas closest to the Reinga-Norfolk ridge system is mirrored in the broscine carabid genus *Mecodema* Blanchard, which includes numerous Northland endemics (Seldon and Leschen 2011). Like *Meonochilus*, *Mecodema* comprises a New Zealand representative of a clade whose sister group is Australian (Roig-Juñent 2000).

## Taxonomic Treatment

Moriomorphini Sloane, 1890: 646 (sensu Liebherr 2011; type genus *Moriomorpha* Castelnau).

Subtribe Moriomorphina Sloane, 1890: 646.

Melisoderides Sloane, 1898: 470 (synonymy Bouchard et al. 2011; type genus *Melisodera* Westwood).

Subtribe Amblytelina Blackburn, 1892: 85 (type genus *Amblytelus* Erichson).

Meonides Sloane, 1898: 470 (NEW SYNONYMY; type genus *Meonis* Castelnau).

Tropopterides Sloane, 1898: 470 (NEW SYNONYMY; type genus *Tropopterus* Solier).

Mecyclothoracitae Jeannel, 1940: 97 (NEW SYNONYMY, type genus *Mecyclothorax* Sharp).

**Tribal Diagnosis.** Mandibular scrobe bearing a seta near anterior margin of excavation, and mesocoxal cavities conjunct, therefore a member taxon of Sylifera Jeannel, 1941(Jeannel 1948); head capsule with frontal grooves extended mediodorsally to area mesad compound eye, not extended as deeply posterad eye as observed in Trechini; apical maxillary palpomere of similar basal breadth to apex of penultimate palpomere, not subulate as in Bembidiini; penultimate maxillary palpomere glabrous or with sparse covering of short setae, not bearing pelage of elongate setae as in Zolini; shaft of third antennomere glabrous, setae limited to apical ring, not with apical half to apical 2/3 of shaft surface bearing elongate setae as in Patrobini and Psydrini (*Psydrus* LeConte, *Nomius* Laporte and *Laccocenus* Sloane); antennal cleaner of “grade C” configuration (Hlavac 1971) with cleaning channel little extended basad clip setae, versus grade B in Psydrini, in which cleaning channel on medial surface of protibia extends 1/3 distance from clip setae towards tibial base; apicolateral margin of elytra continuous, elytral plica an internal ridge that can be viewed laterally, but which is not continuous with apicolateral elytral margin as in Pterostichini; pygidial defensive gland orifice associated with membrane between abdominal tergites VIII and IX, not distinctly fused to either tergite (Deuve 1993); male protarsomeres symmetrically expanded laterally, ventral adhesive setae biseriate; male aedeagal median lobe with closed basal bulb, not with open eudorsal surface such as Apotomini, Broscini, Patrobini, Psydrini, or *Trechodes* Blackburn, nor with open eudextral surface as in Bembidiini and Pogonini (Jeannel 1941); female gonocoxae divided into basal and apical gonocoxites, spermatheca with appended spermathecal gland (Liebherr and Will 1998).

### Key to Genera for Adults of New Zealand Moriomorphini

**Table:** 

1	Anterior margin of labrum straight to broadly and shallowly emarginate; mandibles short to moderately long, the mandibular scrobe bordered ventrally by a broad, horizontal ventral margin, the distance from dorsal mandibular condyle to lateroapical margin of labrum subequal to distance from labral margin to mandibular apex; maxillary lacinial apex acuminate, hooklike; lateral pronotal seta situated near pronotal midlength	2
1’	Anterior margin of labrum deeply emarginate, the depth of emargination 1/3 labral width; mandibles elongate, dorsal and ventral margins of scrobe vertically aligned, the scrobe not bordered ventrally by an extended margin, and the mandibles extended beyond labral margin much more than distance from dorsal condyle to labral margin; maxillary lacinial apex rounded; lateral pronotal seta situated in the apical ¼ of pronotal length, measured along the midline	*Selenochilus* Chaudoir (subtribe Amblytelina)
2	Elytral humerus subangulate to angulate, a small elevated tooth or knob on margin at angle; elytra with eighth interval apically carinate just laterad the seventh stria	3
2’	Elytral humerus rounded, or elytral basal margin reduced, lateral marginal depression terminated at humerus	4
3	Elytra with 0–3 dorsal elytral setae in third interval of third stria (Larochelle and Larivière 2007), fifth interval glabrous; apical visible abdominal ventrite of female with two lateroapical setae each side	*Molopsida* White (subtribe Moriomorphina)
3’	Elytra with 4 dorsal elytral setae on third interval just mesad third elytral stria, fifth interval with single seta near 1/3 elytral length; apical visible abdominal ventrite of female with four lateroapical setae each side plus two subapical setae medially	*Rossjoycea* gen. n. (subtribe Moriomorphina)
4	Head with two supraorbital setae each side; glossal sclerite broadly truncate apically; pronotum with lateral and basal seta present, the lateral seta situated close to lateral depression	*Mecyclothorax* Sharp (subtribe Amblytelina; see Liebherr and Marris 2009)
4’	Head with one supraorbital seta–the posterior–present each side; glossal sclerite medially extended, the lateral margins curved posterad from middle; pronotum with lateral seta present, basal seta absent, the lateral seta situated on lateral area of disc separated from lateral depression	*Meonochilus* Liebherr and Marris (subtribe Amblytelina)

#### Rossjoycea glacialis gen. n. and sp. n.

urn:lsid:zoobank.org:act:081E57E0-40E3-4623-B387-5BE87D78EF0F

urn:lsid:zoobank.org:act:81EDB412-1DE6-4B86-B164-2CE283AFDB72

http://species-id.net/wiki/Rossjoycea_glacialis

##### Type species.

*Rossjoycea glacialis*, sp. n.

##### Diagnosis.

**Figure 7.**
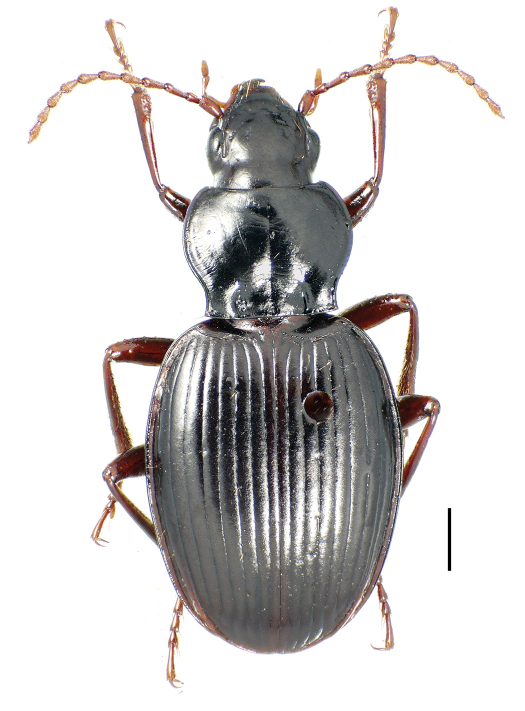
*Rossjoycea glacialis*, female holotype, dorsal view (scale bar = 1.0 mm).

**Figure 8.**
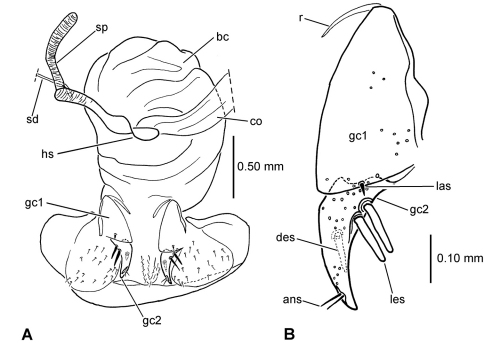
*Rossjoycea glacialis*, ventral view **A** female reproductive tract **B** left female gonocoxite (see Table 1 for abbreviations).

This taxon is amply differentiated from all other taxa in Moriomorphini by the unique combination: 1, small, indistinctly convex eyes, ocular ratio 1.26; 2, cordate, quadrisetose pronotum with impunctate median base (Fig. 7); 3, quadrisetose third elytral interval with a single accompanying seta in basal third of fifth interval; 4, vestigialized metathoracic flight wings; 5, extensively developed isodiametric microsculpture on head and elytra, plus dense subiridescent transverse mesh microsculpture on pronotal disc; 6, apical margin of apical female abdominal ventrite with four setae each side accompanied by a medial pair of subapical setae; and 7, large body size, standardized body length 10.1 mm. The female reproductive tract characters are entirely congruent with those defining membership in subtribe Moriomorphina, including sclerotization of the base of the spermathecal duct forming a helminthoid sclerite at the juncture of the duct, common oviduct and bursa copulatrix (Fig. 8A), and presence of a single lateroapical seta on basal gonocoxite 1 (Fig. 8B). Specimens of the male sex are not known for this taxon, but the cladistic hypothesis predicts that males will possess elongate, parallel-sided parameres as exhibited by males of the other taxa classified in the subtribe Moriomorphina.

##### Description.

*Head capsule* convex across vertex, frontal grooves broad and shallow, broadened medially at frontoclypeal suture, but outlines ill-defined; outer surface of compound eye not extended beyond broad curvature of ocular lobe; 2 supraorbital setae each side, situated mesad a sulcate supraorbital groove that terminates just posterad hind margin of eye; neck transversely depressed, a broad groove connecting 2 posterior supraorbital setae. *Antennae* moderately elongate, antennomere 9 length 2.37× breadth; basal 3 antennomeres glabrous except for dorsal seta on scape, ventral seta on antennomere 2, and apical ring of setae on antennomere 3; apical ¾ of antennomere 4 and all of antennomeres 5–11 setose, the surface sparsely covered with long setae and the glabrous areas of anterior and posterior surfaces covered with well-developed isodiametric sculpticells. *Labrum* 6-setose, anterior margin broadly, shallowly emarginate, the emargination extended posterad 0.10× distance between anteriormost portions of front margin. *Mandibles* robust, moderately short, the extension of left mandible past anterior labral margin 0.7× distance from dorsal condyle to labral anterior margin; dorsal terebral face of mandible with wrinkled surface, the mandibular tip slightly hooked; mandibular scrobe with explanate ventral margin, its flattest and most explanate portion translucent; scrobal seta near anterior margin of excavation, the setal apex extended to margin of scrobe. *Maxillary* stipes unisetose, a single seta present near base; lacinia hooked apically; apex of galea fusiform; maxillary palpomeres glabrous, apical palpomere fusiform. *Labium* with anterior margin of glossal sclerite truncate, bisetose; paraglossae glabrous, short, the distance from base to anterior margin of glossal sclerite greater than length of paraglossal extension beyond glossal sclerite margin. *Mentum* bisetose, lateral lobes deep, breadth 2.5× distance from apical angles of lateral lobes to basal suture; mentum tooth narrowly rounded, with shallow lateral marginal bead and broad, indistinct longitudinal median carina; mentum surface broadly and shallowly depressed laterally; submentum quadrisetose, lateral pair of setae smaller than medial pair. *Pronotum* distinctly cordate, the disc convex relative to deeply incised, linear to C-shaped laterobasal depressions that are separated from the distinctly concave lateral margin by a broad convexity; entire median base of pronotum impunctate with the exception of indistinct punctures associated with transverse wrinkles emanating medially from laterobasal depressions; basal margin without marginal bead medially, but mediobasal margin broadly depressed, indistinctly, longitudinally wrinkled; median longitudinal depression finely, broadly incised on disc; anterior transverse depressions shallow, indistinct medially, absent laterally near rounded, little protruded front angles; lateral marginal depression narrow, margin beaded, the marginal depression equally developed laterally, somewhat narrower and more distinctly incised basally inside concave basolateral margins; hind angles acute, the basal seta equidistant from lateral and basal margins; proepipleura robust, posteriorly expanded dorsoventrally to match the deep anterior portions of elytral epipleura. *Elytra* oviform, lateral margins evenly curved posterad outside pronotal hind angles, the apex broadly curved across the fused elytral halves, the apicolateral margin indistinctly affected by the broad, shallow subapical sinuation; base of elytra laterad broad scutellum depressed, the elytral area between the humeral angles forming a collar where connected to prothorax; scutellar striole shallow and very short; parascutellar seta present; basal marginal groove distinct from halfway point of scutellar interval to angulate humerus; a longitudinal scale-like tooth on basal margin at humerus, the apex of tooth elevated just above juncture of basal groove and elytral lateral margin; all striae impressed from base to apex, intervals moderately convex, the striae minutely punctate basally on elytral disc, smooth laterally and apically; eighth interval distinctly carinate apically immediately laterad stria 7, the carina extended from middle of posterior series of lateral setae to juncture with elytral lateral margin beyond apex of stria 4; interval 3 with 4 setae (pinhole may have obscured fifth seta on right elytron) distributed from 0.20–0.70× elytral length; a single seta in fifth interval near 0.3× elytral length; lateral elytral setae arrayed into an anterior series of 6 setae, followed by a single isolated seta, and a posterior series of 6 setae, these latter 6 much farther one from their neighbor than in the anterior series; lateral marginal depression of elytra wide and horizontal outside the posterior setal series, the elytral margin distinctly and robustly beaded, and the flat marginal depression immediately mesad marginal bead lined with granulate isodiametric sculpticells; elytral plica internal, well developed and visible in lateral view; both apical and subapical seta present; the apices of the elytral halves dissimilar, the right half more extended and more broadly rounded. *Mesepisternum* broadly and distinctly punctate, more than 25 individual punctures present, the surface between punctures lined with distinct isodiametric sculpticells. *Metepisternum* short, anterior margin distinctly longer than lateral margin; metepisternal-metepimeral suture obscure, region of suture broadly depressed and covered with longitudinal wrinkles. *Abdomen* with basal 3 visible ventrites indistinctly punctate laterally; visible ventrites 2–5 with irregular longitudinal wrinkles laterally along posterior margins, and broad, shallow, rounded lateral depressions (best observed a lower magnifications – e.g., 20× – and with oblique lighting). *Legs* thin, elongate; profemur with 1 anterior seta (right side) and anteriorly glabrous (left side); mesofemur bisetose anteriorly; metacoxae bisetose; metafemur bisetose anteriorly; posteroapical surface of protibia and anteroapical surfaces of meso- and metatibiae hirsute, broadly covered with transverse rows of fine, elongate setae in addition to the generalized spike-like longitudinal tibial setae; basal meso- and metatarsomeres with dorsolateral groove, tarsomeres 2 and 3 convex dorsally; fourth tarsomeres lobate, protarsomere with anterior apical lobe length 1.6× mediodorsal tarsomere length, outer apical lobe length 1.4× mediodorsal tarsomere length; mesotarsomere 4 similarly lobate, with anterior (outer) lobe longer than posterior lobe; metatarsomere 4 with length of anterior lobe 0.7× mediodorsal tarsomere length, posterior (inner) lobe 0.6× mediodorsal tarsomere length; fifth tarsomeres with approximately 10 elongate ventrolateral setae in two longitudinal rows, length of longest setae more than 2.5× tarsomere depth at point of setal insertion. *Coloration* somber, head and pronotal disc piceous, the surface subiridescent due to well-developed microsculpture; elytra slightly paler, brunneopiceous; ventral thoracic sclerites piceous, abdominal ventrite rufopiceous mediobasally, lateral reaches of abdomen with piceous cast; femora piceous, tibiae and tarsi paler, rufopiceous to brunneous. *Microsculpture* well developed throughout; vertex of head covered with dense, granulate isodiametric mesh, the sculpticells slightly stretched transversely in dorsal depression of neck; pronotal disc with dense transverse mesh microsculpture, each sculpticell raised, the surface diffracting oblique light rays causing iridescent sheen; discal elytral intervals covered with a mixture of isodiametric and transversely stretched sculpticells that line up in irregular transverse rows, lateral elytral intervals with more transverse mesh, the sculpticells arranged in arched transverse rows; ventral thoracic sclerites with well-developed transverse mesh, abdominal ventrites with dense isodiametric mesh microsculpture.

##### Female reproductive tract.

(n = 1) Bursa copulatrix globose, about as long as broad, common oviduct and spermathecal duct both joined to bursa midventrally (Fig. 8A); spermathecal duct heavily sclerotized basally, the base raised as a ventral crest set at 90° to immediately distal sclerotized duct; spermathecal duct subequal in length to the sinuous spermathecal reservoir, the reservoir of only slightly greater diameter than the duct; spermathecal gland duct entering near base of spermathecal reservoir; basal gonocoxite 1 glabrous except for a single, short seta in the position of the lateroapical series (Fig. 8B); apical gonocoxite 2 with 2 narrow, elongate lateral ensiform setae, their length 0.40× apical gonocoxite length; a single elongate dorsal ensiform seta present mid-dorsally; apical sensory furrow with 2 nematiform setae basad 4 short furrow pegs, the setae articulating at 0.80× gonocoxite length.

##### Holotype.

Female (LUNZ), dissected with body and abdominal ventrites mounted on platens, and dissected reproductive tract in polyethylene vial on pin: Westland N.P. WD / Castle Rocks Hut / 1200 m Chionochloa./ / subalpine scrub 17.i.86 / R.M. Emberson, C.A. Muir // pitfall trap // LUNZ Entomology Research Museum / P O Box 84 Lincoln University / Canterbury, NEW ZEALAND / LUNZ 00009323 // Holotype♀ / Rossjoycea / glacialis / det. J.K. Liebherr 2011 (black-bordered red label).

##### Etymology.

The generic name *Rossjoycea* is a concatenation of the first names of Ross and Joyce Bell, and it honors their work together elucidating the worldwide diversity and relationships of rhysodine carabid beetles. The genus name is to be treated as feminine. The adjectival specific epithet *glacialis* denotes the habitat occupied by this species, with the lone known locality situated between two of the highest arms of the Franz Josef Glacier, in Westland National Park, the South Island, New Zealand.

##### Distribution and Habitat.

The lone female specimen known for this species was collected in a pitfall trap at a site consisting of a flatter area bordered by a steep slope, located immediately above an arm of the Franz Josef Glacier, the South Island, New Zealand. The soil surface was about 70% covered with shrubs or grasses, and about 30% bare ground and rock. The flatter upper area supported an open grassland flora, including the snow tussock grass *Chionochloa* (Poaceae), *Celmisia* (Asteraceae), *Ranunculus* (Ranunculaceae), and *Aciphylla* (Apicaeae). About 60 m elevation lower down the steep slope the habitat changed to one dominated by shrubs, including *Olearia ilicifolia* Hook. f. (Asteraceae), *Hoheria glabrata* Sprague and Summerhayes (Malvaceae), and *Dracophyllum traversii* Hook. f. (Ericaceae) (R.M. Emberson, pers. comm). Field notes made at the time state “Rock-turning not at all successful mainly due to the newness of the terrain” and “Beetle hunting abysmal, perhaps due to the extreme sogginess of the ground (J.W. Early, field notes deposited at LUNZ).”

#### Meonochilus

Liebherr & Marris

http://species-id.net/wiki/Meonochilus

Meonochilus Liebherr and Marris, 2009: 7 (type species *Tarastethus amplipennis* Broun, 1912, by original designation

##### Diagnosis.

Among Moriomorphini, species of *Meonochilus* uniquely display the combination of a single, posterior supraorbital seta, glabrous pronotal basal angle and a lateral pronotal seta that is situated on the lateral portion of the pronotal disc, well separated from the lateral depression in individuals of all species except *Meonochilus bellorum*, sp. n., within which the seta is separated from the lateral depression by a distance equal to the diameter of its articulatory socket. The pronotal median base is distinctly punctate, and the basal and lateral margins of the elytra are joined at a rounded to subangulate humerus. There may be either one or two dorsal elytral setae, the basal seta always present, the apical seta, in the third setal position of interval 3, present only in individuals of *Meonochilus bellorum*. The eighth elytral interval is not subapically carinate, but it may be convex dorsad the subapical sinuation. Possession in the males of apically setose aedeagal parameres, the ventral or right of which also exhibits multiple elongate setae along its ventral margin (Fig. 1D, 2A, 2F, 2G, 6) establishes membership for this genus in subtribe Amblytelina under the cladistic hypothesis presented above.

##### Description.

*Head* *capsule* narrow relative to convex margins of pronotum and inflated elytra; frontal grooves distinctly incised and divergent posterad frontoclypeal suture, ended mesad compound eye, in some individuals shallowly continued posteriorly to shallow groove that marks juncture between ocular lobe and gena. *Antennae* with shafts of basal three antennomeres glabrous, scape with large medioapical seta and shorter ventroapical seta, antennomere 2 with ventroapical seta, and antennomere 3 with apical ring of setae; antennomere 4 with apical ¾ covered with fine setae, and antennomeres 5–11 completely setose; antennal segments submoniliform to moderately elongate, length of antennomere 9 1.6–2.0× maximum breadth (though *Meonochilus spiculatus* deviates by having the antennae longer, with length of segment 9 2.3× its breadth). *Labrum* transverse, anterior margin 6-setose, broadly and shallowly emarginate, the emargination depth no more than 0.07× distance between lateral labral apices. *Mandibles* moderately elongate, their length approximately twice the distance from dorsal condyle to anterior labral margin (mandibles in closed position), apices acute, right mandible with blunt anterior retinacular tooth near apical 1/3 of length, mandibular scrobe bordered ventrally by explanate, translucent margin that is broadest in basal ¼ of length. *Maxillary* stipes 3-setose, a longer ventral seta plus shorter dorsal seta near base and a third seta medially; lacinia with hook-like apex, apex of galea tightly rounded, subacuminate, maxillary palps glabrous except for a short medial seta at apex of penultimate palpomere, and very short trichoid sensilla covering surface of fusiform, dorsoventrally flattened apical palpomere. *Labium* with bisetose glossal sclerite that is narrowed lateroapically; paraglossae glabrous, elongate, their apex extended 1/3 their length beyond apical margin of glossal sclerite; penultimate labial palpomere bisetose on medial edge, apical palpomere apparently glabrous, but covered with very small trichoid sensilla. *Mentum* bisetose, transverse, breadth approximately 3× longitudinal distance from base to apex of lateral lobes; mentum tooth well developed, varying in shape from broadly rounded in *Meonochilus bellorum*, to tightly rounded, and variable within species, to acute in some individuals; mentum setae set in broad shallow lateral depressions; submentum 4-setose. *Pronotum* quadrate to transverse in overall dimensions, the lateral margins broadly convex, basolateral margins either straight or indistinctly sinuate anterad the projected, denticulate hind angles; median base with distinct, isolated punctures, the surface between punctures smooth; mediobasal marginal bead present only in *Meonochilus bellorum*, absent in other species; sparsely punctate to smooth laterobasal depressions distinctly margined posteriorly by well-developed bead that is continuous with basolateral marginal bead inside hind angles; median longitudinal impression distinctly, broadly impressed, anterior transverse impressions broadly shallow, indistinct especially laterally where they are absent near rounded, little-projected front angles; lateral marginal depression narrow in apical half, broader near basal angles, variably translucent depending on sclerotization and melanization of specimen; prosternal process glabrous, unmargined, the posterior surface convex. *Elytra* fused along suture, subglobose, the disc elevated relative to base and lateral margins; scutellum broader than long; parascutellar striole short, deep, isolated from first or sutural stria; parascutellar seta present; basal elytral groove continuous laterad the scutellar interval, continuous with lateral elytral margin at rounded to subangulate humerus; a single dorsal elytral seta in third interval near 1/3 elytral length, except for *Meonochilus bellorum* with a second seta just posterad elytral midlength; lateral elytral setae in two groups, an anterior series of 7 (less commonly 6 or 8) setae, and a posterior series of 6 (rarely 5) setae; apical elytral seta present in apex of seventh stria, subapical seta absent (Fig. 11B) except for *Meonochilus bellorum* with both setae present (Fig. 11A); subapical sinuation shallow and broad viewed dorsally, the elytral plica well developed, internal, not meeting apicolateral elytral margin. *Mesepisternum* punctate, from 8–25 punctures along dorsal portion of surface. *Metepisternum* short, anterior margin longer than lateral margin; metepisternal-metepimeral suture a distinct line in *Meonochilus bellorum*, *Meonochilus rectus* and *Meonochilus spiculatus*, an incomplete line, absent laterally in *Meonochilus eplicatus* and *Meonochilus placens*, and a broad indistinct depression in *Meonochilus amplipennis*. Metathoracic flight wings vestigial, the wing rudiment not extended beyond posterior margin of metanotum. *Abdomen* with visible ventrites 3–5 bearing 1–2 distinct longitudinal impressions laterally each side; apical ventrite of male with 2 larger setae, one each side (incorrectly reported as 4 setae, 2 each side by Liebherr and Marris, 2009: 9), except for *Meonochilus bellorum* within which the 2 larger setae are accompanied by 2 shorter subapical setae medially; apical ventrite of female with four setae, two each side, plus 4 medial subapical setae arranged trapezoidally with the shorter parallel anterad. *Legs* with generalized setation; profemur glabrous anteriorly; mesofemur bisetose anteriorly; mesotibia and metatibia with diffuse lateroapical setal fields, the field arranged in 4 irregular transverse rows on mesotibia, in 4–7 irregular rows on metatibia (least developed in *Meonochilus bellorum*, within which transverse rows may be indicated by only 1 or 2 setae); metacoxa bisetose, the two setae lateral; metafemur bisetose anteriorly; tarsomeres glabrous dorsally; tarsomeres I–IV broadened apically, the dorsal surface broadly convex with fine lateral grooves each side; female protarsomere 4 with broadly elongate apical lobes, lateroapical lobe length 2× dorsomedial length of tarsomere, medioapical lobe length 3× dorsomedial length; male protarsomere 4 lobes of similar breadth but longer, lateroapical lobe 3× dorsomedial tarsomere length, medioapical lobe 4× dorsomedial length; male protarsomeres I–III with biseriate, ventral squamose setae, 5–6 paired setae on I, 4–5 on II, and 4 on III, ventral surface of each lobe on protarsomere 4 covered each side with 4–5 longitudinal series of long silky setae, the apical setae extended to apical ¼ of tarsomere 5; claws of protarsomere 5 acuminate, ½ length of tarsomere; mesotarsomeres 1–5 relatively similar in configuration to those of protarsus, though each segment shorter overall; mesotarsomere 4 lobate, lateral lobe longer, length 3× mediodorsal tarsomere length. *Microsculpture* generally reduced; vertex shiny, obsolete transverse mesh microsculpture traceable over portions of medial surface (best developed in *Meonochilus amplipennis*); pronotal disc shiny, microsculpture generally obsolete, small areas of reduced transverse mesh microsculpture may be visible in some individuals; pronotal median base glossy, smooth between distinct punctures; laterobasal pronotal depression glossy, indistinct isodiametric sculpticells may be visible in the irregular wrinkles of surface; elytral microsculpture most evident, though still much reduced, in association with striae and punctures, elytral intervals generally glossy, though elongate transverse mesh microsculpture is present on intervals in *Meonochilus amplipennis*,and in some individuals of *Meonochilus eplicatus*.

##### Male genitalia.

Aedeagus with basal bulb closed, a variously developed sagittal crest present, orientation in repose such that anatomical right side is ventral (Fig. 2A, 2B, 2F, 2G, 16); right or ventral paramere narrow, length ranging from 0.50–0.80× distance from parameral articulation with median lobe to external face of apex; left or dorsal paramere broader in basal half and narrowed apically, its length 0.5–1.0× distance from parameral articulation to external face of apex; both parameres setose, the right paramere bearing setae broadly distributed over apical half of ventral edge and surrounding parameral tip, the left with 2–3 larger apical setae accompanied by 0–3 smaller setae.

##### Female reproductive tract.

Gonocoxae bipartite, the basal gonocoxite bearing a lateroapical series of 1–3 setae (always more than 1 seta on at least one gonocoxa), and a series of much smaller setae on the mesal surface; apical gonocoxite with 2 lateral ensiform setae, a single dorsal ensiform seta that is near the dorsomedial surface of the coxite, and an apical sensory furrow bearing 2 nematiform setae; bursa copulatrix columnar in shape, without helminthoid sclerite near the junction with the common oviduct; spermatheca with appended spermathecal gland; membranous ramus present mesad base of basal gonocoxite 1.

##### Distribution and Habitat.

Species of *Meonochilus* are restricted geographically to the North Island of New Zealand. Though specific forest habitats recorded for the various species may differ, nearly all specimens of *Meonochilus* with recorded ecological data have been collected from ground-level microhabitats. These collecting events include activity-based pitfall trapping, and discovery of specimens in terrestrial situations such as under logs or stones, in leaf litter, or along a stream. Though recorded collections include only a few specifically mentioning collecting at night, present evidence suggests that *Meonochilus* beetles do not climb on vegetation or vertical tree trunks at night, and thus they differ in their behavior from species of *Molopsida* spp. wherein individuals can be found walking on the bark of upright trees as well as downed logs after dark.

##### Key to Species for Adults of *Meonochilus* Liebherr and Marris

**Table:** 

1	Elytra concolorous, brunneous to piceous; pronotal base margined laterally posterad laterobasal depression, without marginal bead medially (Fig. 9B, 9C, 10); a single dorsal elytral seta each side near basal fourth of third interval; standardized body length 4.9–6.7 mm	2
1’	Elytra with lateral and apical margins flavous, contrastedly paler than brunneous disc, the paler marginal areas encompassing intervals 6–8 near humerus, 7–8 and marginal depression near midlength, and all of apex except sutural interval that may be darker, rufous (Fig. 9A); two dorsal elytral setae each side, the anterior near basal ¼, the posterior near elytral midlength; standardized body length 3.8–4.2 mm	1. *Meonochilus bellorum*, sp. n.
2	Pronotal base broad, maximum pronotal width to basal pronotal width ratio 1.23–1.41; elytral base broad, humeri extended laterally (Fig. 9C, 10)	3
2’	Pronotal base constricted, basolateral margins distinctly sinuate anterad denticulate hind angles, maximum pronotal width to basal pronotal width ratio 1.44–1.49; elytra oviform, basolateral margins narrowly rounded posterad humeri (Fig. 9B)	2. *Meonochilus spiculatus*, sp. n.
3	Elytral striae 1–5 deep, stria 6 shallower, stria 7 obsolete to irregularly discontinuous; standardized body length smaller, 4.9–6.1 mm	4
3’	Elytral striae 1–7 deep, continuous from elytral base to apex, all elytral intervals distinctly convex; standardized body length larger, 6.1–6.7 mm	4. *Meonochilus amplipennis* (Broun)
4	Pronotum more quadrate, maximum pronotal width to median pronotal length 1.29–1.34 (Fig. 9C, 10B); basal and lateral elytral margins evenly, broadly rounded at humerus	5
4’	Pronotum more transverse, maximum pronotal width to median pronotal length ratio 1.39–1.42 (Fig. 10C); juncture of basal and lateral elytral margins tightly rounded to subangulate at humerus	6. *Meonochilus placens* (Broun)
5	Suture between metepimeron and metepisternum incomplete, suture evident medially but effaced laterally; internal sac of male aedeagal internal sac unarmed, membranous (Fig. 16A)	5. *Meonochilus eplicatus* (Broun)
5’	Metepimeron and metepisternum separated by distinct, complete suture; internal sac of male aedeagal internal sac with large, spikelike flagellum (Fig. 2G)	3. *Meonochilus rectus*, sp. n.

**Figure 9.**
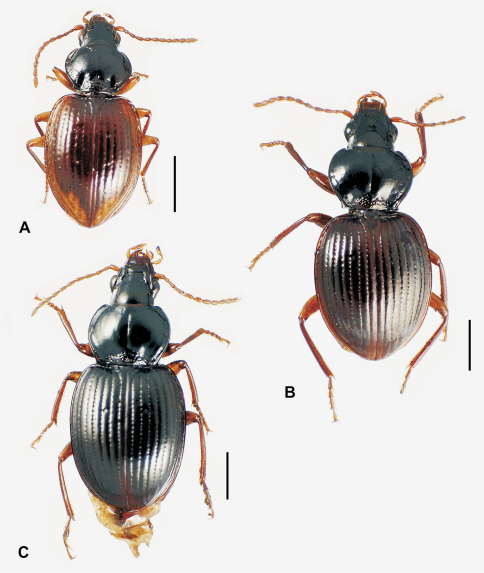
*Meonochilus* spp., dorsal view **A** *Meonochilus bellorum* **B** *Meonochilus spiculatus* **C** *Meonochilus rectus* (scale bars = 1.0 mm).

**Figure 10.**
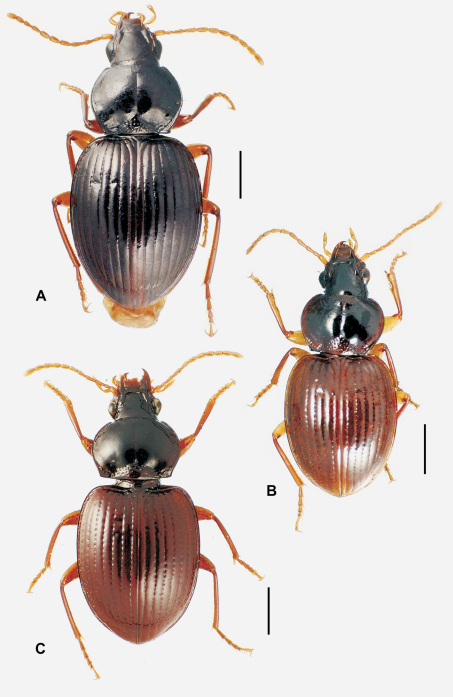
*Meonochilus* spp., dorsal view **A** *Meonochilus amplipennis* **B** *Meonochilus eplicatus* **C** *Meonochilus placens* (scale bars = 1.0 mm).

**Figure 11.**
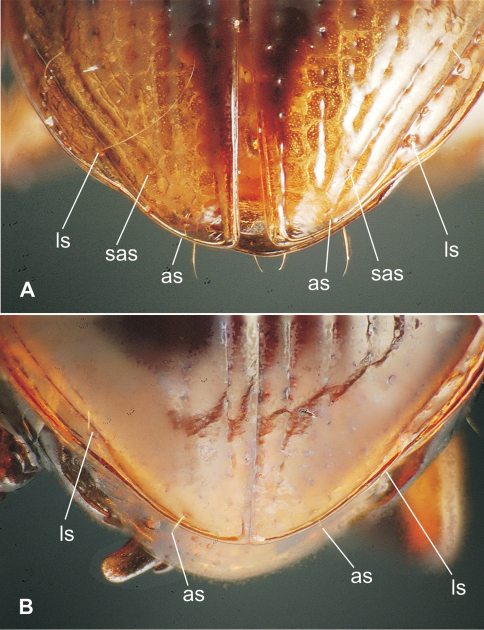
Elytral apex, dorsal view, illustrating position of apical setae (as), subapical setae (sas), and posterior seta of posterior lateral setal series (ls) **A** *Meonochilus bellorum* female **B** *Meonochilus spiculatus* male.

**Figure 12.**
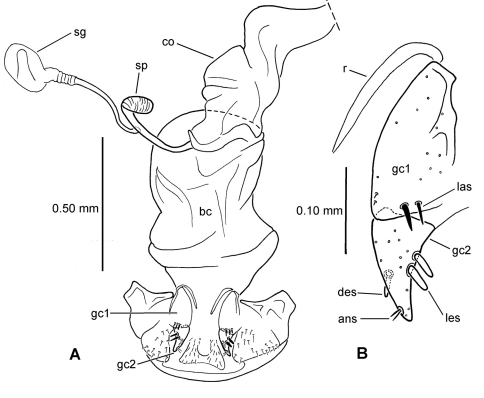
*Meonochilus bellorum*, ventral view **A** female reproductive tract **B** left female gonocoxite (see Table 1 for abbreviations).

#### 1. Meonochilus bellorum sp. n.

urn:lsid:zoobank.org:act:AF87FB29-809C-4390-B72E-F2BA94C2A033

http://species-id.net/wiki/Meonochilus_bellorum

##### Diagnosis.

Differing from all other species of the genus in many respects, the most obvious being the bicolored elytra, with the outer intervals flavous versus median intervals dark brunneous to piceous (Fig. 9A). Individuals are also much smaller than those of other species, with standardized body length 3.8–4.2 mm. Other characters that confirm the diagnosis include: 1, pronotum with complete basal marginal bead; 2, elytra with 2 dorsal setae in third interval; 3, both apical and subapical elytral setae present (Fig. 11A); 4, mesepisternum with approximately 8 large punctures on dorsal portion of surface, versus 12–25 punctures in the other species; 5, metepisternum separated from metepimeron by distinct suture, visible externally as a complete, depressed line; 6, male aedeagus with elongate right paramere (Fig. 1D, 1E); and 7, internal sac of male aedeagus with sinuous, flagellum and flagellar sheath apically on left side (Fig. 1E).

##### Male Genitalia.

(n = 1). Aedeagal median lobe broad, robust, with broadly expanded sagittal crest and broadly rounded and downturned apex (Fig. 1D), the apex turned rightward in ventral view (Fig. 1E); internal sac elongate, membranous except for apical flagellar complex; flagellum defined by position ventrad gonopore (Fig. 1D), and consisting of shorter flagellar sheath and longer flagellum associated with left side of apex; apex of sac to right of flagellar complex covered with crescent-shaped roll of membrane that is covered with short, stout, spikelike microtrichia (brush sclerite; Lindroth 1976); right (ventral in repose position) paramere elongate, length 0.8× distance from parameral articulation point on median lobe to outer surface of median lobe apex, 6 setae on parameral ventral edge, 3 setae at tip, and 3 setae on the dorsal margin; left paramere broadest medially, with attenuate apex, length subequal to distance from parameral articulation to lobe apex, a single longer seta at the very narrow parameral tip, a shorter seta on ventral margin of paramere near tip.

##### Female Reproductive Tract.

(n = 2) Bursa copulatrix columnar, distance from gonocoxal bases to apex 1.5× bursal breadth; spermathecal duct joining bursa ventrally at juncture with common oviduct, the spermathecal duct directed rightward from common oviduct-bursal juncture (Fig. 12A); spermathecal duct length subequal to length of spermathecal reservoir, reservoir a curved tube of diameter similar to spermathecal duct; spermathecal gland duct base at juncture between spermathecal reservoir and spermathecal duct; basal gonocoxite 1 with lateroapical series of 2(3) setae, and medioapical series of 2–6 setae extended from mesal portion of apex basad along apical half of mesal surface (Fig. 12B); apical gonocoxite 2 with 2 long, narrow lateral ensiform setae, and 1 dorsal ensiform seta on dorsomedial surface; apical sensory furrow bearing 2 nematiform seta at 0.88× coxite length.

##### Holotype.

Male (NZAC): NEW ZEALAND ND / Omahuta For., Kauri / Sanct., Pukekohe Stm / tk 300m 30.I.1995 / Larivière/Larochelle // Taraire-kauri for. / Wet muddy / streambank / Under dead leaves. // NZ Arthropod / Collection, NZAC / Private Bag 92170 / AUCKLAND / New Zealand (yellow label) // HOLOTYPE ♂ / Meonochilus / bellorum/ det. J.K. Liebherr 2011 (black-bordered red label) // ♂ habitus photo / J.K. Liebherr 2011.

##### Paratypes.

ND: Matarua For., Waioku Coach Rd. Tk., Wekaweka entry, wet roadside/wet *Caldcluvia* for./litter, 35°33.91'S, 173°36.22'E, 400 m el., 09-ii-1995, Larivière/Larochelle (NZAC, 1); Omahuta For., Kauri Sanctuary, Pukekohe Str., Taraire-kauri for., wet muddy streambank, under dead leaves, 35°14.46'S, 173°37.74'E, 300 m el., 30-i-1995, Larivière/Larochelle (NZAC, 2); under *Elastostema*/veg. debris, streambank, 35°14.46'S, 173°37.74'E, 300 m el., 31-i-1995, Larivière/Larochelle (NZAC, 2), Trounson Kauri Pk., Kaihu litter, 35°43.29'S, 173°38.94'E, 19-i-1972, Ramsay (NZAC, 1), kauri-podocarp hdwd., wet leaves/flood debris/forest stream, 250 m el., 03-07-xii-1984, Newton/Thayer (FMNH, 1); Warra Warra St. For., [Warawara St. For], ex leaves from stream in bush, 35°22.40'S, 173°17.65'E, 10-x-1974, Dugdale (NZAC, 2).

##### Etymology.

The species epithet *bellorum* is a genitive plural commemoration of Ross and Joyce Bell’s ground-breaking, indeed log-breaking research into the biology of “rhysodid” beetles. The species for commemoration was chosen based on: 1, collection of most of the specimens by André Larochelle and Marie-Claude Larivière, former denizens of the climax maple forest biome; and 2, the disparate mosaic of plesiomorphic and apomorphic characters displayed by beetles comprising *Meonochilus bellorum*.

##### Distribution and Habitat.

**Figure 13.**
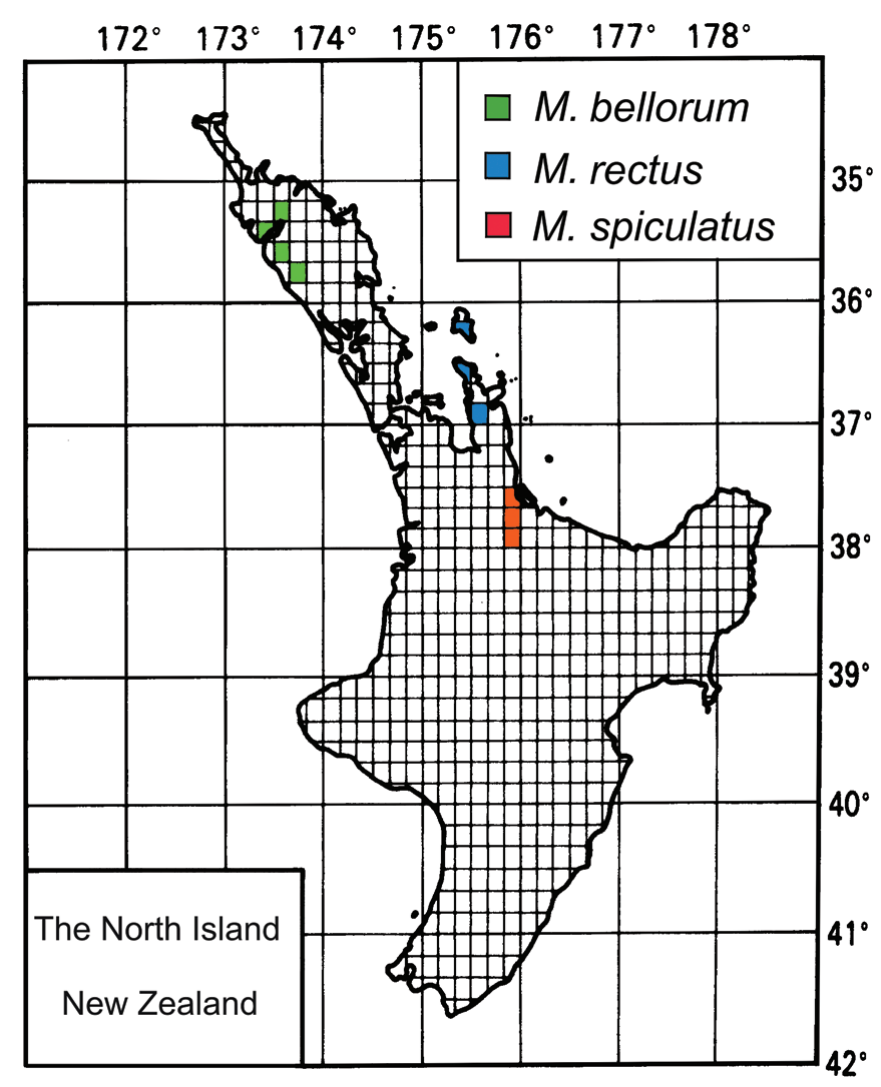
Distribution map for *Meonochilus* spp.

This species is known to occupy only a limited distributional range in western Northland, with collecting localities centered on Hokianga Harbor (Fig. 13). Most beetles of *Meonochilus bellorum* have been collected from very wet microhabitats, including in flood debris and among leaves from a stream, under vegetal litter and debris on a streambank, and in litter along a wet roadside. All collecting records are in October, or from December to February.

#### 2. Meonochilus spiculatus sp. n.

urn:lsid:zoobank.org:act:6E9CFCDF-2190-4B96-9EA5-424BAE633883

http://species-id.net/wiki/Meonochilus_spiculatus

##### Diagnosis.

Individuals of this species are best told by the narrow pronotal and elytral bases, resulting in a medially constricted body shape (Fig. 9B). The suboviform elytra have deep, distinctly punctate striae and convex discal intervals with glossy surfaces. The antennae are moderately elongate, not submoniliform, with 9^th^ antennomere length 2.3× maximum breadth. The male aedeagal internal sac bears a long, stout, spikelike flagellum, and the median lobe apex terminates in a narrow, symmetrically rounded bulblike tip (Fig. 2F). This species exhibits a distribution parapatric to *Meonochilus amplipennis* in Bay of Plenty (Fig. 13, 15), but *Meonochilus spiculatus* can be easily diagnosed by its smaller body size–standardized body length 5.7–5.9 mm versus 6.1–6.9 mm–and through comparison of male aedeagal apices, *Meonochilus amplipennis* being characterized by an asymmetrical apicoventrally directed expansion (Fig. 2A).

##### Male Genitalia.

(n = 2). Aedeagal median lobe with narrowly elongate sagittal crest, shaft of lobe narrow and straight in apical half, terminated in a symmetrically rounded apex; internal sac with spikelike flagellum and closely appressed flagellar sheath (Fig. 2F), the sac narrowly extended beyond flagellar point of insertion; a rolled crescent of membrane bearing short, stout spikelike microtrichia present immediately to right of flagellar base (brush sclerite; Lindroth 1976); ventral, right paramere short, length about ½ distance from point of parameral articulation to external face of apex, ventral and dorsal edges subparallel, apex rounded, 6–8 setae on ventral margin (those closer to base shorter), adjacent to 9–14 large setae arrayed on rounded apex, and with 2–5 very short setae in apical half of dorsal edge; dorsal left paramere subconchoid, broad basally with apex narrowly rounded, little extended, and 2 larger setae on dorsal surface at tip, and 2 smaller setae just basad.

##### Female Reproductive Tract.

**Figure 14.**
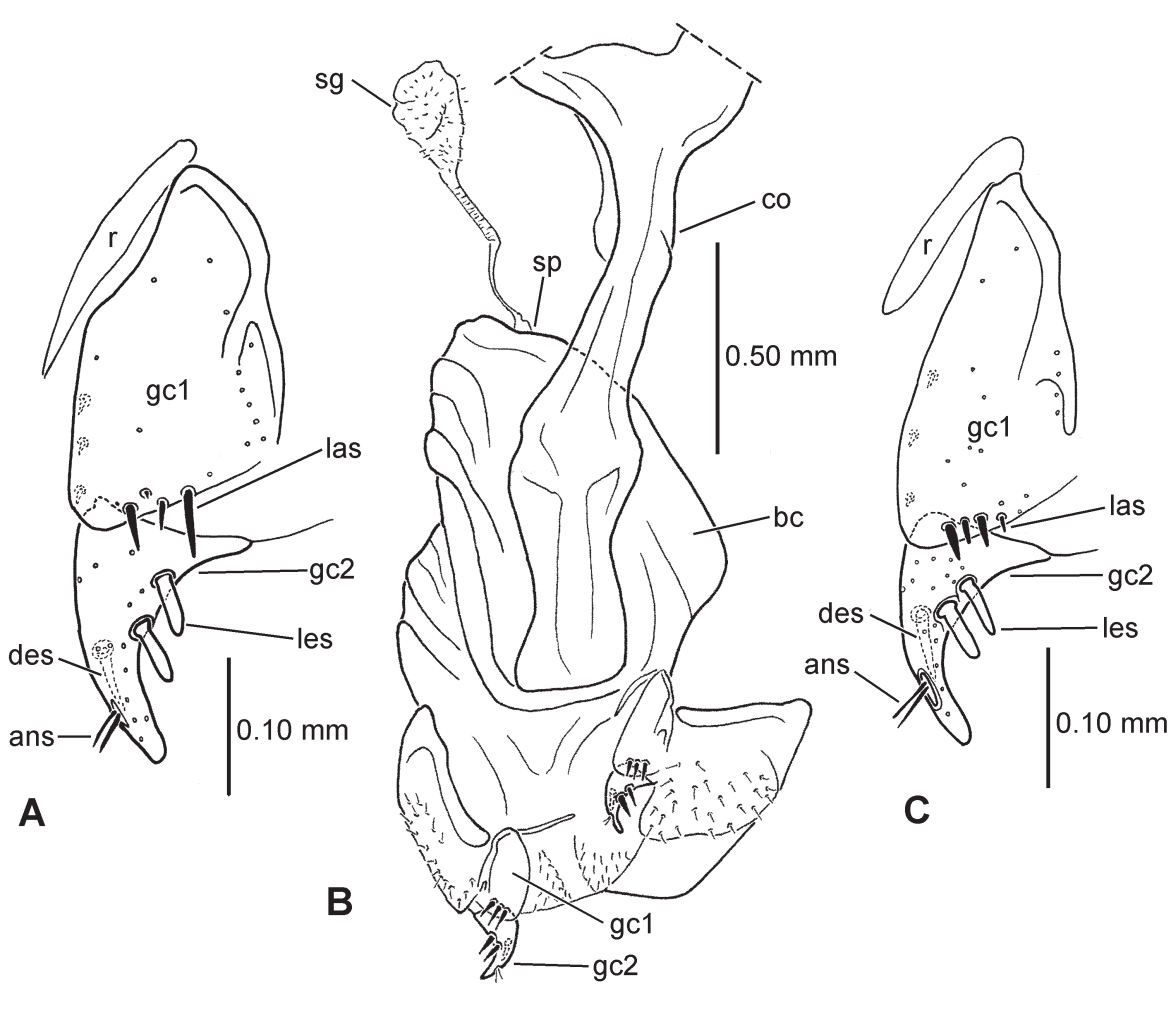
Female reproductive tract structures, *Meonochilus* spp., ventral view **A** left gonocoxite, *Meonochilus spiculatus* **B** female reproductive tract, *Meonochilus rectus* **C** left gonocoxite, *Meonochilus rectus* (see Table 1 for abbreviations).

(n = 1) Bursa copulatrix narrowed apically to bulbous, appressed spermatheca, distance from gonocoxal bases to apical spermatheca 1.6× greatest width of bursa at juncture with common oviduct (Fig. 4A); common oviduct broadest at juncture with bursa, heavily membranous based on dense staining with Chlorazol Black; spermatheca a broadly rounded protuberance situated on apex of bursa, the spermathecal gland duct entering directly into the protuberance; spermathecal gland duct approximately twice as long as apical ductile reservoir; basal gonocoxite 1 with lateroapical series of 3 setae, 2 larger and 1 smaller, each side, and medioapical series of 3–5 setae extended along apical half of dorsomedial surface (Fig. 14A); apical gonocoxite 2 with 2 broad lateral ensiform setae, and 1 dorsal ensiform seta on dorsal surface; apical sensory furrow bearing 2 nematiform seta at 0.70× coxite length.

##### Holotype.

Male (NZAC), dissected and mounted on 3-line platen, genitalia enclosed in polyethylene vial on pin: Kaimai Range / Matamata / Waikato, N. Is., N.Z. // Coll / A.E. Brookes, / 12-4-1941. / A.E. Brookes / Collection / NZ Arthropod Collection / [bar code] / NZAC04007286 // HOLOTYPE ♂ / Meonochilus / spiculatus / det. J.K. Liebherr 2011 (black-bordered red label).

##### Paratypes.

BP: Kaimai, 37°40.21'S, 175°51.80'E, 01-iii-1948, Brookes (NZAC, 1); Mt. Te Aroha, litter, 37°31.56'S, 175°43.77'E, 21-x-1967, Watt (NZAC, 1), summit, 37°32.10'S, 175°44.56'E, 950 m el., i-2002, Thorpe (AMNZ, 8), in forest at night, 5-i-2003, Thorpe (AMNZ, 2), in litter 92/6, 27-ii-1992, Dugdale (NZAC, 2), under logs and stones, 22-i-2002, Thorpe (AMNZ, 2), under wood on ground, 08-iv-2001, Thorpe (AMNZ, 1); Okauia, Matamata, E, 37°47.26'S, 175°50.35'E, 29-xii-1947, Brookes (NZAC, 1).

##### Etymology.

The species epithet *spiculatus* is a Latin adjective with male ending denoting the apically pointed, spike-like flagellum of the male aedeagal internal sac.

##### Distribution and Habitat.

This species is restricted to the Kaimai Range (Fig. 13) in the northwest edge of Bay of Plenty district (Crosby et al. 1976). Specimens labeled with concise elevations are all from higher elevations, especially on Mt. Te Aroha. This species and *Meonochilus amplipennis* are sympatric at Te Aroha, however *Meonochilus amplipennis* is recorded from lower elevations (740–950 m el.) whereas *Meonochilus spiculatus* records are only from the summit at 950 m el. Specimens have been collected in the forest at night, in litter, and under logs, stones, and wood on the ground.

#### 3. Meonochilus rectus sp. n.

urn:lsid:zoobank.org:act:B3B1A69B-D519-4DC2-8055-44D04BB4ECCE

http://species-id.net/wiki/Meonochilus_rectus

##### Diagnosis.

Individuals of this species look like a broad-bodied version of *Meonochilus spiculatus*, with the broadly based, subparallel elytra exhibiting deep, distinctly punctate striae and convex, glossy intervals (Fig. 9C). The antennae are not as elongate as those of *Meonochilus spiculatus*, with the ninth antennomere length 1.8–2.0× its maximum breadth. Like *Meonochilus spiculatus*, the internal sac of the male aedeagus bears a spikelike flagellum (Fig. 2F, 2G), however the flagellum of *Meonochilus rectus* males is stouter and more sinuously curved, the aedeagal median lobe is broader (Fig. 2G), and it terminates in a downwardly expanded apex. Standardized body length 5.4–5.7 mm.

##### Male Genitalia.

(n = 1). Aedeagal median lobe with basally deep sagittal crest, the lobe shaft broad and robust with ventral surface recurved before downturned and broadly rounded apex (Fig. 2G); internal sac elongate, with stout spikelike flagellum inserted on ventral surface about half the distance from ostium to apex of apical membranous lobe; flagellar sheath closely appressed to flagellum, length about half the length of flagellum; convex roll of sac membrane extended on right side of flagellar articulation toward sac apex, the roll covered with broad shingle-like sculpticells, the exposed shingle edges lining the distal edges of sculpticells (brush sclerite; Lindroth 1976); ventral right paramere parallel-sided and apically rounded, length equal to ½ distance from parameral articulation to external face of apex, 10 long setae lining the apical half of ventral edge, 12 long setae arrayed on convex parameral apex, and 6 long, thinner setae in apical half of dorsal edge; dorsal left paramere broad basally, subconchoid, apex narrowly rounded, 2 long setae on dorsal surface near tip, with 2 short setae immediately basad to those.

##### Female Reproductive Tract.

(n = 1) Bursa copulatrix broadest medially, extended to broadly rounded apex that constitutes spermatheca (based on insertion of spermathecal gland duct), overall bursal plus spermathecal length 1.1× maximal bursal breadth (Fig. 14B); common oviduct broadest distad juncture with ventral surface of bursa; spermathecal gland duct short, length approximately equal to basal stem of spermathecal gland, the narrow duct base joining bursal apex at short, stump-like protuberance; basal gonocoxite 1 with lateroapical series of 3–4 setae, a single additional seta basal to the series of 4 on one side of the lone dissected female specimen, 3–4 smaller setae along apical half of dorsomedial surface (Fig. 14C); apical gonocoxite 2 with 2 broad lateral ensiform setae and 1 dorsal ensiform seta on dorsal surface; apical sensory furrow bearing 2 nematiform setae at 0.70× coxite length.

##### Holotype.

Male (NZAC): NEW ZEALAND CL / Great Barrier I / Mt. Hobson Tk. / 24 Mar- / 27 Mar 1978 // J.C. Watt + / J. Murcer / Pit trap 9 // N Z Arthropod Collection / [bar code] / NZAC04007299 // HOLOTYPE ♂ / Meonochilus / rectus / det. J.K. Liebherr 2011 (black-bordered red label).

##### Paratypes.

CL: Great Barrier Island, Mt. Hobson, leaf litter, RL805, 36°11.27'S, 175°24.69'E, 500 m el., 17-xii-2003, Leschen (AMNZ, 1), pitfall trap, 9-xi-17-xii-2003, Early (AMNZ, 2), 17-xii-2003-07-iv-2004, Early (AMNZ, 1), Te Paparahi, pitfall trap, G1, 35°26.07'S, 174°23.43'E, iii-2002, Warren (AMNZ, 1), Windy Hill, pitfall trap, 36°18.12'S, 175°32.11'E, 200 m el., 30-iv-11-vi-2003, Parsons (AMNZ, 1); Mt. Moehau, under log, 36°32.05'S, 175°23.97'E, 765 m el., 19-iii-1980, Holloway (NZAC, 1); Tapu-Coroglen Rd., Tk. to Crosbies, 36°59.42'S, 175°35.26'E, 13-xi-1996, Gleeson (NZAC, 1).

##### Etymology.

The species epithet *rectus*–Latin adjective with male ending–denotes the short, straight and parallel to slightly converging basolateral margins of the pronotum present immediately anterad the pronotal hind angles.

##### Distribution and Habitat.

The distribution of *Meonochilus rectus* includes the Coromandel Peninsula and Great Barrier Island to the north (Fig. 13). This range lies disjunctly north of the Kaimai Range distribution of the sister species, *Meonochilus spiculatus*, suggesting that a vicariant barrier separating the mountains of Coromandel and Bay of Plenty facilitated allopatric speciation. Nonetheless, eustatic sea level changes that isolated Great Barrier Island from the mainland have not resulted in speciation of the island population. Most specimens of this species have been collected via pitfall trapping, with singletons found in leaf litter and under a log. Localities range in elevation from 200–765 m el.

**Figure 15.**
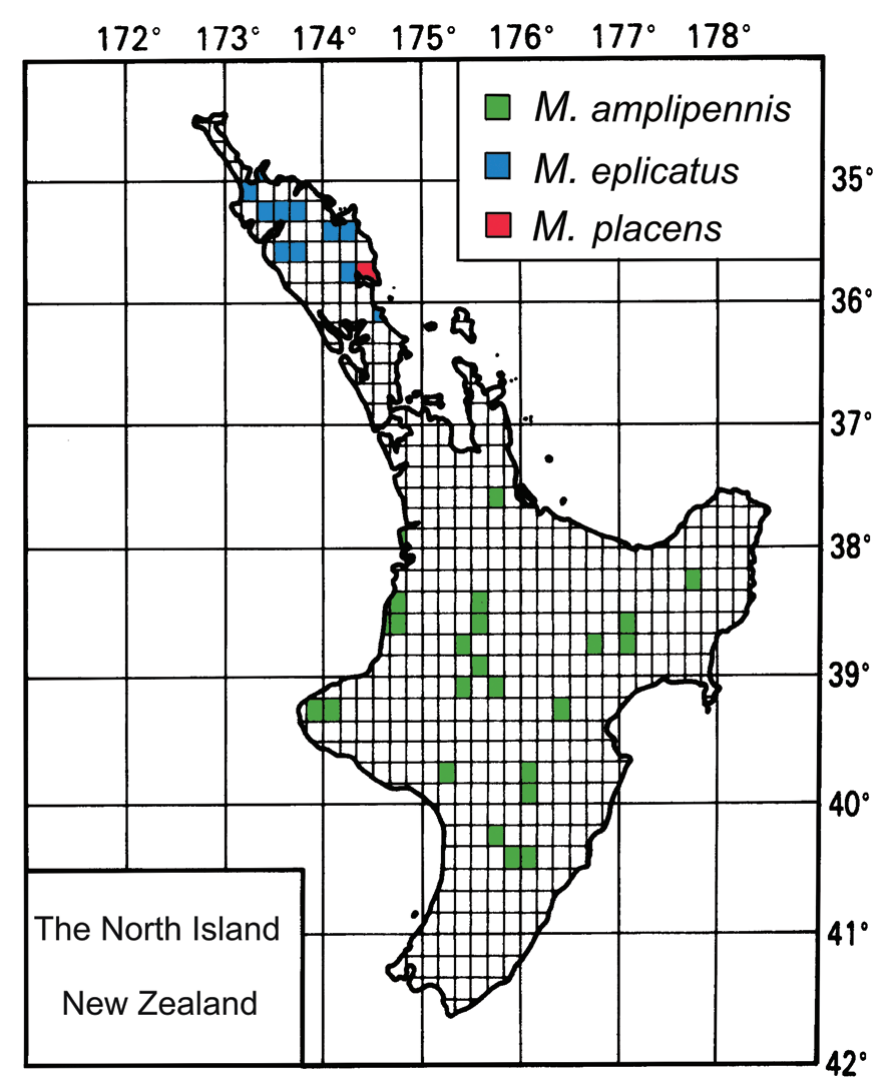
Distribution map for *Meonochilus* spp.

#### 4. Meonochilus amplipennis

(Broun)

http://species-id.net/wiki/Meonochilus_amplipennis

urn:lsid:zoobank.org:act:

Tarastethus amplipennis amplipennis Broun, 1912: 386.
Molopsida amplipennis amplipennis , Britton, 1940: 477.
Mecyclothorax amplipennis amplipennis , Larochelle and Larivière, 2001: 61.
Meonochilus amplipennis amplipennis , Liebherr and Marris, 2009: 10.
Tarastethus amplipennis labralis Broun, 1912: 387 (NEW SYNONYMY).
Molopsida amplipennis labralis , Britton, 1940: 477.
Mecyclothorax amplipennis labralis , Larochelle and Larivière, 2001: 62.
Meonochilus amplipennis labralis , Liebherr and Marris, 2009: 10.

##### Diagnosis.

This is the largest-bodied *Meonochilus*; standardized body length 6.1–6.7 mm. In addition, individuals of this species have elytral striae 1–8 deeply impressed, with stria 7 as deep and as punctate as stria 6. The elytral striae are the least punctate of all *Meonochilus* spp., with the punctures restricted to the deepest portions of the striae and associated with minimal lateral strial expansion (Fig. 10A). The discal elytral intervals are covered with traceable, elongate transverse sculpticells. The male aedeagal median lobe terminates in a ventral expansion, and the internal sac bears a spiculate lobe on the apicodextral surface (Fig. 2A, 2B), however no flagellum is present.

##### Male Genitalia.

(n = 9). Aedeagal median lobe with short, thin sagittal crest, apical half of shaft straight to recurved ventrally, terminated in an explanate ventral expansion (Fig. 2A); internal sac densely covered with microspicules, these densest on the dorsal surface, though largest and most sclerotized in two fields, one on the right surface, and one on a more apically positioned, rounded lobe; ventral, right paramere short, length ½ distance from parameral articulation to outer face of apex, broadest near midlength and so appearing subtriangular in lateral view, apical half of paramere setose, the ventral surface with 6–8 thinner setae immediately adjacent to 16–20 larger, thicker setae lining the rounded apex, 4–6 smaller setae on the dorsal edge; dorsal left paramere subconchoid, apex narrowly rounded, 2–3 larger setae at tip interspersed with up to 6 small setae.

##### Female Reproductive Tract.

(n = 1) Bursa copulatrix columnar, distance from gonocoxal bases to apex 1.67× maximal breadth; spermatheca an appressed button-like reservoir on dorsal surface of bursa (Fig. 4B); spermathecal gland duct entering spermatheca on distal end, the duct length subequal to length of apical ductile reservoir of gland; common oviduct broadly joined to bursa just distad base of gonocoxites; basal gonocoxite 1 with apicolateral series of 2–4 setae, 3–4 setae arrayed along apical half of mesal surface (Fig. 5C); apical gonocoxite 2 with 2 broad lateral ensiform setae and 1 dorsal ensiform seta on dorsal surface; apical sensory furrow bearing 2 nematiform setae at 0.75× gonocoxite length.

##### Types.

For *Tarastethus amplipennis*, lectotype male (BMNH), mounted on unmarked white platen, labeled: Type (round, red-bordered label) // 3170. // New Zeal. / Broun Coll. / Brit. Mus. / 1922 – 482. // Raurimu. / Jany. 1910. // Tarastethus / amplipennis ♂ // Lectotype ♂ / Tarastethus / amplipennis / Broun, 1912 / det. J.K. Liebherr 2011 (black-bordered red label). Paralectotype female (BMNH) identically labeled except for: Paratype (round yellow-bordered label) [first label] // … // Paratype / Tarastethus / amplipennis Broun / E.B. Britton [fifth label] // Paralectotype ♀ … [bottom label] (black-bordered red label). For *Trechus amplipennis labralis*, lectotype female (BMNH), identically mounted and labeled as *Amblytelus amplipennis* lectotype except for: var. A. / labrum. [fourth label] // Type / Tarastethus / amplipennis Broun / labralis Broun / E.B. Britton [fifth label] // Lectotype ♀ / Tarastethus / amplipennis labralis / Broun, 1912 / det. J.K. Liebherr 2011 (black-bordered red label).

##### Non-type Material.

BP:Mt. Te Aroha, 37°32.10'S, 175°44.52'E, 740–950 m el., 07-v-2003, Thorpe (AMNZ, 3). GB: "Auckland (sic)", Mt. Maungapohatu, 38°35.08'S, 177°07.08'E, 914–1219 m el., 03-iii-1971, Townsend (NZAC, 3); Aiawhana Station, Tarndale Rd., 38°13.07'S, 177°47.71'E, 762 m el., 28-ii-1971, Townsend (NZAC, 1); Huiarau Range, Putaihinu Ridge, 38°35.47'S, 177°06.47'E, 1158 m el., 02-iii-1971, Townsend (NZAC, 3), Putaihinu Ridge, S end, 38°36.74'S, 177°05.86'E, 914 m el. 02-iii-1971, Townsend (NZAC, 4); Uwewera N.P., Huiarau summit, pittrap/*Nothofagus*/Horopito/tree fern, 38°37.96'S, 177°01.90'E, 923 m el., 14–19-ii-1994, Larivière/Larochelle (NZAC, 8), Ngaputahi, 10 km S, Okahu Rd. end, under log/open wet broadleaf for., 38°40.57'S, 176°48.57'E, 500 m el., 16-ii-1994, Larivière/Larochelle (NZAC, 2), Ta Taita a Makoro Camp, pittrap/low *Nothofagus* for./litter, 38°41.01'S, 177°03.40'E, 700 m el., 14–18-ii-1994, Larivière/Larochelle (NZAC, 1). HB: Kaweka For. Pk., Black Birch Ra. Tk., N side, Kaweka Rd., under log, 39°19.00'S, 176°26.36'E, 1000 m el., 04-iii-1996, Larivière/Larochelle (NZAC, 1), Kaweka Flats Tk.-N Boulder Str. Tk. jct., 39°13.92'S, 176°24.95'E, 1000 m el., 03-iii-1996, Larivière/Larochelle (NZAC, 2); Kaweka Range, Little’s Clearing to Black Birch Range, along stream, 39°16.89'S, 176°26.25'E, 13-xi-1988, Townsend (NZAC, 1), Ngahere Hut, baited pit trap, 39°17.44'S, 176°24.87'E, 980 m el., 20-27-xii-1983, Watt (NZAC, 1); Makahu Saddle, Ngahere Loop Tk., under log/mtn. beech for., 39°17.29'S, 176°24.45'E, 1000 m el., 22-ii-02-iii-1996, Larivière/Larochelle (NZAC, 1). RI: Copper Creek, 14-iv-1957, Townsend (NZAC, 1); Mangawhero R., Kakatahi Str., Te Hue Rd., 39°40.34'S, 175°19.99'E, 09-i-1989, Townsend (NZAC, 1); Ruahine St. For. Pk., Rangitane Range, Kawhatau Base, pittrap/Horopito/*Melicytus*/tree fern for., 39°46.82'S, 176°02.22'E, 800 m el., 25-ii–iii-1994, Larivière/Larochelle (NZAC, 3), Rangitane Rd. end, Colenso Trig. Tr., 39°45.26'S, 176°02.73'E, 850–1000 m el., 26-ii-1994, Larivière/Larochelle (NZAC, 1), Short’s Tk.-Limestone Rd. end jct., under stone/moist for./Horopito/treefern, 39°59.34'S, 176°01.14'E, 750 m el., 03-i-1994, Larivière/Larochelle (NZAC, 2). TK: Lucy'S, Gully S of New Plymouth, 39°12.08'S, 173°57.26'E, 02-x-2007, Chogstall/Townsend (JITC, 1); Mt. Egmont, 23-ii-1929, Brookes (NZAC, 1), Dawson Falls, 39°19.53'S, 174°06.42'E, 03-iv-1957, Townsend (NZAC, 1), Manganui R., Curtis Falls, 39°17.96'S, 174°06.65'E, 880 m el., 02-i-1990, Townsend (NZAC, 2), Ihaia Tk., under stone/wet broadleaf for./mud flats, 39°19.71'S, 173°59.20'E, 500 m el., 20-iii-1998, Larivière/Larochelle (NZAC, 2), Oaonui Tk., 39°18.56'S, 173°59.64'E, 28-ii-1981, Townsend/Townsend (JITC, 2), Ngatoro Tk., under log/wet broadleaf for./mud flats 39°14.70'S, 174°07.43'E, 650 m el., 19-iii-1998, Larivière/Larochelle (NZAC, 1), North Egmont, pitfall trap, 39°16.11'S, 174°05.66'E, 470 m el., 14-xii-2003–12-i-2004, Stringer (AMNZ, 2), Holly Hut, pit trap, 39°15.90'S, 174°02.84'E, 950 m el., 27-xi-1975, Walker (NZAC, 1), under *Uncinia rubra*, 39°15.90'S, 174°02.84'E, 950 m el., 28-xi-1975, Dugdale (NZAC, 1), Pouakai Range, Dover Tk. Under branch/wet broadleaf for., 39°13.65'S, 173°58.33'E, 500 m el., 21-iii-1998, Larivière/Larochelle (NZAC, 1), Pouakai Hut, under log/*Senecio eleaguifolius*, 39°14.12'S, 174°02.26'E, 1280 m el., 03-xii-1975, Walker (NZAC, 1), Stratford Mountain House, mossy logs/trees at night/kamahi-totara, 39°18.34'S, 174°07.30'E, 850 m el., 26-xii-1994, Emberson/Syrett (LUNZ, 1), Stratford Plateau, under rock/*Senecio* scrub, 39°18.46'S, 174°06.01'E, 1100 m el., 27-xii-1985, Emberson/Syrett (LUNZ, 1). TO: Awakino, N Manganui Saddle, 38°38.97'S, 174°39.46'E, 26-iv-1986, Townsend (NZAC, 1); Hauhungaroa Range, west side, 38°42.97'S, 175°26.91'E, 20-xi-1965, Townsend (NZAC, 2); Mahoenui, Mangaorongo Rd., 38°34.99'S, 174°50.00'E, 31-x-1977, Townsend (NZAC, 1); Moerangi, Waituhi Saddle, 38°51.80'S, 175°32.65'E, 09-x-1979, Dugdale (NZAC, 1); Pihanga, Hinemihi'S, Tk., 39°00.27'S, 175°46.76'E, 22-xi-1992, Townsend (NZAC, 1); Pureora For. Pk., Bog Inn Tk., under log/wet broadleaf-podocarp for., 38°34.90'S, 175°38.16'E, 22-xii-1995, Larivière/Larochelle (NZAC, 3), Link Tk., 38°34.88'S, 175°38.00'E, 700 m el., 25-xii-1997, Larivière/Larochelle (NZAC, 10), Mt. Pureora Tk., Link Rd., halfway, under log/wet podocarp broadleaf for., 38°33.80'S, 175°38.10'E, 20-xii-1995, Larivière/Larochelle (NZAC, 6), lower 1/2, under log/wet Rimu for., 38°34.20'S, 175°38.08'E, 20-xii-1995, Larivière/Larochelle (NZAC, 7), start of tk., pit trap/wet Rimu for., 38°35.04'S, 175°38.27'E, 23-x-1995–3-i-1996, Larivière/Larochelle (NZAC, 1), Mt. Titiraupenga, Link Rd., under log/wet podocarp broadleaf for., 38°30.67'S, 175°41.60'E, 24-xii-1995, Larivière/Larochelle (NZAC, 3), Waihora Rd., Waihora Tk., 38°38.47'S, 175°39.75'E 19-xii-1995, Larivière/Larochelle (NZAC, 5); Rangitoto St. For., Mangatutu Sce. Res., under log, 38°20.16'S, 175°27.38'E, 22-iii-1999, Paquin/Dupérré (NZAC, 1); Raurimu, 39°07.05'S, 175°23.12'E, 26-xii-1940, Clarke (AMNZ, 2), 27-xii-1940, Clarke (AMNZ, 2); Tongariro N.P., Lake Rotopounamu, under log/mixed podocarp-broadleaf for., 39°01.41'S, 175°43.97'E, 24-xii-1991, Emberson/Syrett (LUNZ, 1). WA: Waewaepa Range, 40°26.08'S, 176°03.35'E, 29-iii-1957, Cumber (NZAC, 1). WN: Balance Bridge, Cross Rd. [Pahiatua, N], litter, 40°25.47'S, 175°51.77'E, 213 m el. 23-viii-1983, McColl (NZAC, 1); Manawatu Gorge, under log, 40°19.27'S, 175°48.71'E, 25-iv-1956, Townsend (NZAC, 1). WO: Herangi Range, Moeatoa Sce. Res., pittrap/wet broadleaf for./gully stream, 38°22.51'S, 174°44.41'E, 122 m el., 20-viii–24-ix-2000, Larivière/Larochelle (NZAC, 1); Mangatoa Sce. Res., pittrap/wet broadleaf for./gully stream, 38°25.66'S, 174°42.75'E, 191 m el., 23-x–03-xii-2000, Larivière/Larochelle (NZAC, 1); Mt. Karioi, litter, 37°51.79'S, 174°48.03'E, 11-x-1981, Butcher (NZAC, 1); Whakapatiki Stream, Upper Awakino R., 38°36.95'S, 174°45.57'E, 26-iv-1986, Townsend (JITC, 2); Whareorino St. For., Leitch’s Clearing, 38°25.89'S, 174°46.63'E, 25-iv-1986, Nunn (LUNZ, 1).

##### Nomenclatural Note.

Broun (1912) proposed the variety “labralis” for a single specimen of *Meonochilus amplipennis* from Raurimu that had been collected with the two other types of the species. This specimen curiously lacks all six setae across the anterior labral margin, though the dimensions and microsculpture of the labrum appear as in the syntopic, nominate specimens. As all other attributes do not allow diagnosis of this variety, his validated name *Meonochilus amplipennis labralis* (Larochelle and Larivière 2001) is considered a junior synonym of the nominate name, and the specimen with the glabrous labrum considered an infraspecific mutant.

##### Distribution and Habitat.

The most geographically widespread species of *Meonochilus*, *Meonochilus amplipennis* is found in forested areas of the North Island, from Mt. Te Aroha on the north to northern Wellington and Waiararapa Districts on the south (Fig. 15). Individuals have been collected from moist gully and streambank habitats at lower elevations (100–200 m el.) to higher elevation forest habitats (900–1300 m el.). The montane forests include a variety of formations, including *Nothofagus* forest, Horopito/*Melicytus* tree fern forest, wet rimu forest, kamahi-totara forest, and *Senecio* scrubland. All specimens save one have been collected from ground-level microhabitats, by pitfall trapping, or under logs or stones. The lone semi-arboreal record was collected at night from a mossy log in kamahi-totara forest.

*Meonochilus amplipennis* is parapatrically distributed with *Meonochilus spiculatus* at Mt. Te Aroha (Fig. 13, 15), with *Meonochilus amplipennis* found at lower elevations from 740–950 m on the mountain, whereas *Meonochilus spiculatus* is restricted to the summit at 950 m.

**Figure 16.**
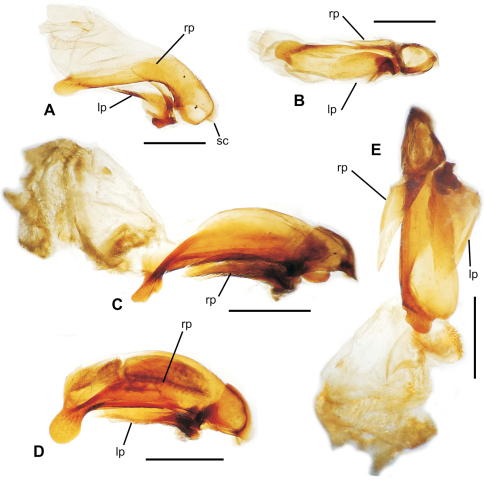
Male aedeagal median lobe and associated parameres, *Meonochilus* spp. **A** *Meonochilus eplicatus*, right lateral view, internal sac everted **B** *Meonochilus eplicatus*, ventral view, internal sac everted **C** *Meonochilus placens*, right lateral view, internal sac everted, lobe apex either abraded and worn, or deformed (compare to Fig. 16D) **D** *Meonochilus placens*, right lateral view, internal sac not everted **E** *Meonochilus placens*, ventral view, internal sac everted (see Table 1 for abbreviations; scale bars = 0.5 mm).

#### 5. Meonochilus eplicatus

(Broun)

urn:lsid:zoobank.org:act:

http://species-id.net/wiki/Meonochilus_eplicatus

Tarastethus eplicatus Broun, 1923: 675.
Molopsida eplicatus , Britton, 1940: 477.
Mecyclothorax eplicatus , Larochelle and Larivière, 2001: 62.
Meonochilus eplicatus , Liebherr and Marris, 2009: 10.

##### Diagnosis.

Individuals of this species can be told by the broadly and moderately convex elytral intervals, and distinctly punctate striae (Fig. 10B)–though the punctures are less distinct than in *Meonochilus rectus* or *Meonochilus spiculatus* (Fig. 9B, 9C)–with the intervals covered with elongate transverse microsculpture that varies based on sex. Males exhibit glossy elytral intervals with remnants of microsculpture in the striae, whereas females exhibit more broadly distributed sculpticells over portions, or the entire surfaces of the intervals. The pronotal hind angles are denticulate with the basolateral margins on each side converging slightly immediately anterad the angles. The basolateral marginal bead is broadly convex inside the hind angles. The body is narrower than in *Meonochilus placens*, with the pronotal base more constricted, and the elytral humeri less expanded laterally (Fig. 10B, 10C). The male aedeagal median lobe apex is broadly rounded and slightly asymmetrically expanded ventrally (Fig. 16A), but not nearly to the degree as observed in males of *Meonochilus placens* (Fig. 16D). As in *Meonochilus amplipennis* and *Meonochilus placens*, the male aedeagal internal sac is membranous. Standardized body length 4.9–5.9 mm.

##### Male Genitalia.

(n = 4). Aedeagal median lobe with variable sagittal crest, from broad and deep to shallow and short, median lobe shaft with straight ventral margin, apex slightly downturned and broadly rounded (Fig. 16A); internal sac membranous, densely covered with fine microspicules, more heavily pigmented roll of membrane situated to right of gonopore; ventral right paramere stalked, length 0.60–0.66× distance from parameral articulation to outer face of apex, base thinner and apical half inflated, apex broadly rounded, apical half of ventral margin to tip lined with 7–15 setae, dorsal portion of tip with only several shorter setae accompanying ventral series, and dorsal edge glabrous or with 1 seta; dorsal left paramere broad, conchoid, length equal to 0.75–0.80× distance from parameral articulation to external face of apex, rounded apex with 2–3 larger setae interspersed with 2–3 small setae (Fig. 16B).

##### Female Reproductive Tract.

**Figure 17.**
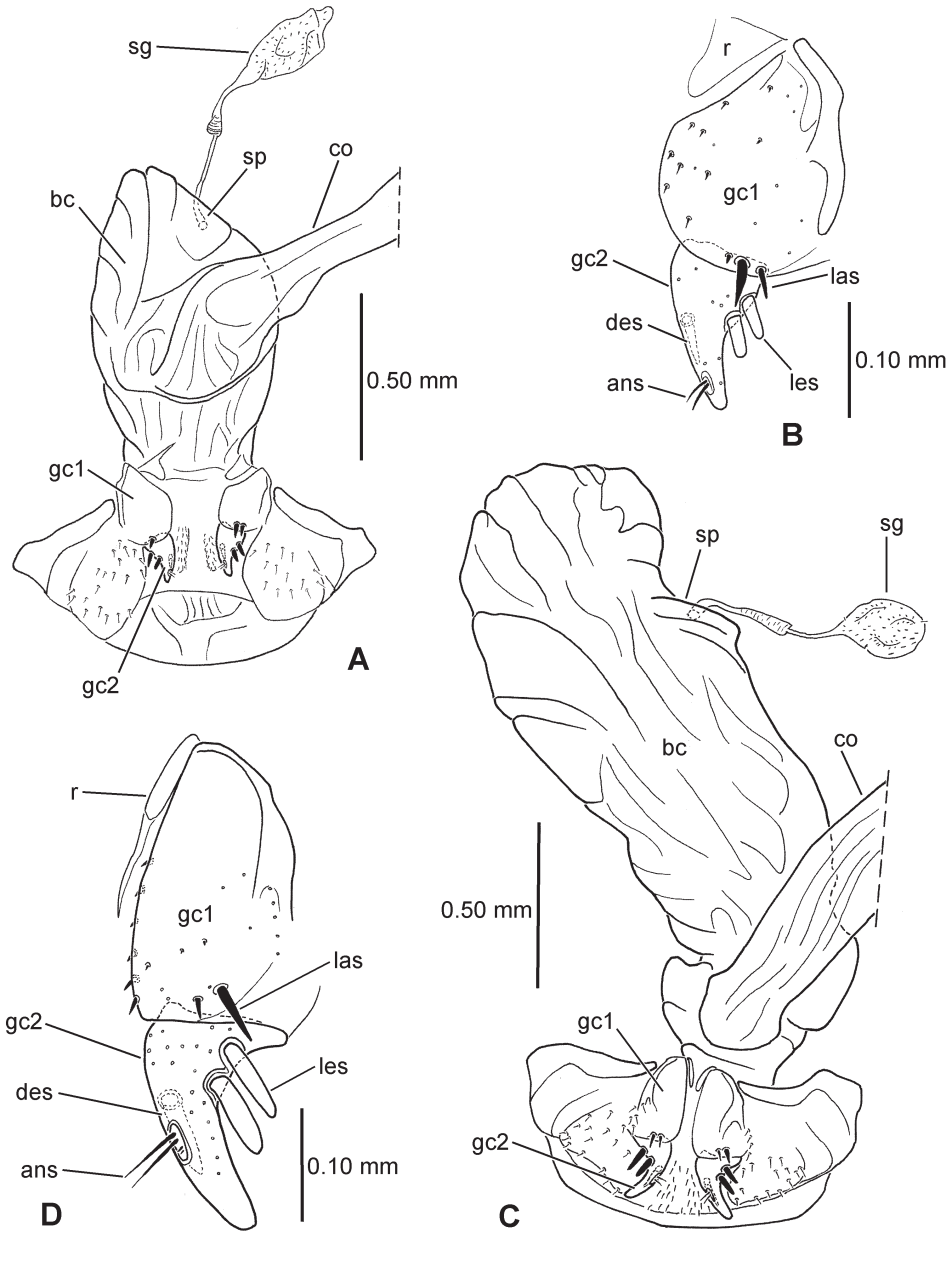
Female reproductive tract structures, *Meonochilus* spp., ventral view **A** female reproductive tract, *Meonochilus eplicatus* **B** left gonocoxite, *Meonochilus eplicatus* **C** female reproductive tract, *Meonochilus placens* **D** left gonocoxite, *Meonochilus placens* (see Table 1 for abbreviations).

(n = 2) Bursa copulatrix elongate, narrowed apically to rounded apex, distance from between gonocoxal bases to apex 1.55× maximal bursal breadth (Fig. 17A); spermathecal gland duct entering bursa on left side of apex, the spermatheca not discernible among the membranous rolls of cuticle comprising bursal apex; spermathecal gland duct a short connective between stem of ductile reservoir and elongate, expanded basal tube that enters bursal wall; common oviduct ventrally joined to bursa, expanded to greatest breadth immediately distad juncture; basal gonocoxite 1 with lateroapical series of 1–2 setae, and mesal series of 8–10 setae in apical half of mesal surface (Fig. 17B); apical gonocoxite 2 broadly subtriangular, with 2 broad lateral ensiform setae and 1 dorsal ensiform seta on dorsomedial surface; apical sensory furrow bearing 2 nematiform setae at 0.80× coxite length.

##### Types.

Lectotype female (BMNH), mounted on unmarked white platen, labeled: Type (round, red-bordered label) // Pakarau. / 19.5.18. // New Zealand / Broun Coll. / Brit. Mus. / 1922 – 482. // Tarastethus / eplicatus // Lectotype ♀ / Tarastethus / eplicatus / Broun, 1923 / det. J.K. Liebherr 2011 (black-bordered red label). Paralectotype female (BMNH), identically mounted and labeled, except for: Paratype (round yellow-bordered label) [first label] // Kaiangaroa / 12.5.1918 [second label] // … Paralectotype ♀ … [fifth label].

##### Non-type Material.

ND: Kara, Whangarei, 35°43.22'S, 174°18.01'E, ix-1942, Fairburn (NZAC, 1); Kawakawa, Whangae Stream, 35°22.64'S, 174°03.88'E, 14-xi-1994, Townsend (JITC, 1); Mangamuka Gorge Walkwy., pittrap/wet broadleaf for., 35°11'S, 173°27'E, 350 m el., 27-v–29-vi-1999, Larivière/Larochelle (NZAC, 1); Mangawhai Gorge, under stone at night, 36°04.09'S, 174°35.02'E, 25-i-1948, Pritchard (NZAC, 2); Ngaiotonga Sce. Res., litter, 35°19.05'S, 174°15.36'E, 20-i-1972, Ramsay, (NZAC, 1); Puketi St. For., litter, 35°13.52'S, 173°44.51'E, 21-i-1972, Ramsay (NZAC, 2); Rangiahua, 35°16.03'S, 173°37.18'E, 01-xi-1966, Cumber (NZAC, 1); Waimatenui, 35°36.72'S, 173°43.42'E, 06-i-1942, Clarke (AMNZ, 1), 09-iii-1961, Cumber (NZAC, 1); Waipoua St. For., litter, 35°36.57'S, 173°32.06'E, 07-xii-1961, Townsend (NZAC, 1), sifted litter, 80/121, 35°36.57'S, 173°32.06'E, 25-xi-1980, Kuschel (NZAC, 1); Waitangi St. For., litter and decayed wood, 81/117, 35°15.19'S, 174°00.56'E, 02-xi-1981, Kuschel (NZAC, 1).

##### Distribution and Habitat.

Of the three Northland species, *Meonochilus eplicatus* as currently circumscribed exhibits the broadest geographic range (Fig. 15). Though genitalic characters do not support dismemberment of this species, the variation in elytra microsculpture should be studied further with additional material from across the range. Specimens collected to date have been from ground-level microhabitats, collected via pitfall trapping in wet broadleaf forest, from leaf litter, or under stones.

#### 6. Meonochilus placens

(Broun)

urn:lsid:zoobank.org:act:

http://species-id.net/wiki/Meonochilus_placens

Tropopterus placens Broun, 1880: 28.
Tarastethus placens , Sharp, 1886: 373.
Molopsida placens , Britton, 1940: 477.
Mecyclothorax placens , Sherley et al., 1999: 299.
Meonochilus placens , Liebherr and Marris, 2009: 10.

##### Diagnosis.

Individuals of this species are best told by the very transverse pronotum, maximum pronotal width/median pronotal length of 1.39–1.42 in association with the very broad pronotal base and broadly based, subparallel elytra (Fig. 10C). Along with individuals of *Meonochilus eplicatus*, the elytral striae are not so deep, and thus the elytral intervals are correspondingly less convex. However, as in individuals of *Meonochilus spiculatus* and *Meonochilus rectus*, the elytral intervals are glossy, without evident microsculpture. The male aedeagal median lobe exhibits an apex that is broadly, ventrally expanded (Fig. 16C, 16D), and a membranous internal sac with a spiculate basal lobe on the right side (Fig. 16C, 16E). Standardized body length 5.7–6.1 mm.

##### Male Genitalia.

(n = 4). Aedeagal median lobe with deep sagittal crest, shaft broad and robust, ventral margin straight before very broadly rounded and downturned apex; internal sac densely covered with microspicules, the spicules on a rounded, ventroapical lobe somewhat larger; ventral right paramere elongate, length equal to 0.75× the distance from point of parameral articulation to external face of apex, sides parallel and apex subtruncate, apical half of ventral surfaced lined with 7–18 long setae, only 1 subapical seta on truncate apex, and 0–1 small setae near apex on dorsal edge; dorsal left paramere broad, elongate, length equal to 0.8× distance from parameral articulation to outer apical face, ventral margin recurved and dorsal margin straight resulting in a “wooden shoe-like” shape, apex (i.e. toe) bluntly rounded, 3–4 setae on ventral surface of apex.

##### Female Reproductive Tract.

(n = 1) Bursa copulatrix exceedingly elongate, distance from gonocoxal bases to bursal apex 3.2× maximal bursal breadth, the membranous bursal surface thin throughout based on minimal staining with Chlorazol Black; spermatheca present as broad, bulbous wart-like structure situated subapically on left surface of bursa (Fig. 17C); spermathecal gland duct entering spermatheca via elongate, expanded base; common oviduct joined to bursa immediately distad gonocoxal bases; basal gonocoxite 1 with lateroapical series of 2 setae, 1 larger, 1 smaller, and mesal series of 5–6 setae along apical half of mesal surface (Fig. 17D); gonocoxite 2 broadly spatulate apically, with 2 acuminate lateral ensiform setae and 1 dorsal ensiform seta along dorsomedial margin; apical sensory furrow bearing 2 nematiform setae near midlength of gonocoxite 2, the nematiform setal insertions at 0.55× gonocoxite length.

##### Types.

Lectotype male (BMNH), mounted on plain white platen, labeled: Manaia // var. / 61 // New Zealand / Broun Coll. / Brit. Mus. / 1922 – 482. // Lectotype ♂ / Tropopterus / placens / Broun, 1880 / det. J.K. Liebherr 2011 (black-bordered red label) // Meonochilus / placens ♂ (Broun) photo / det. J.K. Liebherr 2010. Paralectotype female (BMNH), mounted and labeled as lectotype, except for: 61. [blue label] // Type (round red-bordered label) // ... // Paralectotype ♀ [sixth label].

##### Non-type Material.

ND: Bream Head, Whangarei, 35°50.68'S, 174°34.85'E, 01-vi-1957, Watt(?) (NZAC, 1), under stone, 310 m el., 08-vii-1957, Watt (NZAC, 1); Mt. Manaia, Whangarei Heads, 35°49.11'S, 174°31.02'E, 14-iii-1970, May (NZAC, 1).

##### Distribution and Habitat.

Though this species was first collected by Thomas Broun, and subsequently collected by Charles Watt, it has never been found anywhere except in the Whangerei Heads area north of Whangarei Harbor, Northland (Fig. 15). The restricted distribution of this species reiterates Watt’s (1977) concerns about the importance of conserving type localities of New Zealand insects in order to maintain biodiversity. The sister group relationsip between *Meonochilus placens* and the more widespread western Northland *Meonochilus eplicatus*, suggests that Whangarei Heads has been a peripherally isolated locality at which the population of *Meonochilus placens* speciated from populations comprising the geographically more widespread and morphologically more variable *Meonochilus eplicatus*. In this instance, then, Whangarei Heads is both a center of speciation and a presently limited biodiversity hotpot.

The lone ecological note for the five known *Meonochilus placens* specimens involves collection of one under a stone; thus this is apparently a terrestrially bound species.

## Supplementary Material

XML Treatment for Rossjoycea glacialis

XML Treatment for Meonochilus

XML Treatment for Meonochilus bellorum

XML Treatment for Meonochilus spiculatus

XML Treatment for Meonochilus rectus

XML Treatment for Meonochilus amplipennis

XML Treatment for Meonochilus eplicatus

XML Treatment for Meonochilus placens

## Appendix 1

### Appendix 1

**Table:** List of taxa included in the cladistic analysis of Moriomorphini, excluding *Rossjoycea glacialis*, sp. n. and *Meonochilus* spp., and institutional codens where dissected specimens are deposited. All specimen labels indicate determination by JK Liebherr, 2011.

**Taxon**	**Institution**
*Trechus obtusus* Erichson	CUIC
*Raphetis darlingtoni* Moore	MCZ
*Selenochilus piceus* (Blanchard)	CUIC, LUNZ
*Meonis semistriatus* Sloane	CUIC
*Meonis uncinatus* Baehr	MCZ
*Mecyclothorax lophoides* (Chaudoir)	CUIC
*Mecyclothorax montivagus* (Blackburn)	CUIC
*Mecyclothorax punctipennis* (Macleay)	CUIC
*Neonomius laevicollis* (Sloane)	MCZ
*Tropopterus duponcheli* Solier	FMNH, MCZ
*Amblytelus curtus* (F.)	CUIC
*Molopsida pretiosa* (Broun)	NHMB
*Theprisa australis* (Castelnau)	FMNH
*Moriomorpha* sp. “Dunoon, NSW”	MCZ
*Moriomorpha* sp. “Sherbrook, V”	MCZ
